# MicroRNA-378a-3p is overexpressed in psoriasis and modulates cell cycle arrest in keratinocytes via targeting BMP2 gene

**DOI:** 10.1038/s41598-021-93616-8

**Published:** 2021-07-09

**Authors:** Wipasiri Soonthornchai, Pattarin Tangtanatakul, Kornvalee Meesilpavikkai, Virgil Dalm, Patipark Kueanjinda, Jongkonnee Wongpiyabovorn

**Affiliations:** 1 Division of Immunology, Department of Microbiology, Faculty of Medicine, Center of Excellence in Immunology and Immune-Mediated Diseases, Chulalongkorn University, Rama 4 Road, Bangkok, 10330 Thailand; 2 Department of Transfusion Medicine and Clinical Microbiology, Faculty of Allied Health Sciences, Chulalongkorn University, Bangkok, 10330 Thailand; 3 Division of Mycology, Department of Microbiology, Faculty of Medicine, Chulalongkorn University, Bangkok, 10330 Thailand; 4 Division of Clinical Immunology, Department of Internal Medicine, Erasmus University Medical Center, Rotterdam, The Netherlands

**Keywords:** Immunology, Diseases, Medical research, Molecular medicine

## Abstract

Psoriasis is a chronic autoimmune skin disease driven by dysregulations at the cellular, genomic and genetic levels. MicroRNAs are key mediators of gene expression regulation. However, how microRNAs control the pathogenesis of psoriasis is still unclear. Here, we reported a significant up-regulation of miR-378a-3p (miR-378a) in skin biopsies from active psoriatic lesions while it was down-regulated after treatment with methotrexate or narrow-band ultraviolet B phototherapy. Using the keratinocyte in vitro model, we showed that miR-378a disturbed the cell cycle progression, causing cell cycle arrest at G1 phase. Transcriptomic analysis of keratinocytes with miR-378a overexpression and depletion revealed several important biological mechanisms related to inflammation and tight junction. Target mRNA transcript assessed by luciferase assay identified bone morphogenetic protein 2 as a novel target gene of miR-378a. These findings offer a mechanistic model where miR-378a contributes to the pathogenesis of psoriasis.

## Introduction

Psoriasis is a chronic inflammatory skin disease characterized by complex immunopathogenic processes in which a variety of cells, including keratinocytes, dermal vascular cells, and immune cells have reportedly been implicated^1^. The disease typically manifests as well-demarcated erythematous plaques with adherent silvery scales caused by an accumulative infiltration of effector immune cells in both dermis and epidermis as well as an aberrant proliferation and diminished terminal differentiation of keratinocytes^1–3^. Psoriasis results from a multifaceted interaction of genetic, epigenetic, immunological, and environmental factors. Cumulative evidence shows that keratinocytes play an active role in initiation and maintenance of psoriatic lesions by producing chemokines, cytokines, and antimicrobial peptides, which further attract and activate immune cells to accelerate the inflammatory mechanism in the lesions^4,5^. Despite various therapeutic approaches that exist for the treatment of psoriasis, conventional treatments, such as methotrexate (MTX) and narrow-band ultraviolet B phototherapy (NB-UVB), are still widely used because of their cost-effectiveness in reducing proliferation and inducing apoptosis of hyperplasia^6–9^. However, the complex mechanism that explains the effectiveness of MTX and NB-UVB in psoriasis, especially genetic regulation, still needs to be clarified. Besides, several vital cytokines, such as tumor necrosis factor-α (TNF-α), interleukin-17A (IL-17A), and interferon-γ (IFN-γ), have been shown to involve in the pathogenesis of psoriasis^10^. These cytokines have been identified as therapeutic targets, leading to the possible development of various new targeted therapeutic drugs. Again, the complex interplay between cellular and molecular mechanisms caused by these factors in the pathogenesis of psoriasis has not yet been fully understood.

MicroRNA (miRNA) is crucial in regulating various biological processes through its ability to control gene expression at a post-translational process by decreasing mRNA stability via RNA-induced silencing complex-mediated degradation of targeted mRNA^11^. A number of microRNAs have explicitly been implicated in multiple cellular mechanisms, such as angiogenesis, proliferation, differentiation, apoptosis, organ development, and signal transduction^11,12^. Nonetheless, dysregulation of miRNA expression has frequently been observed in psoriasis^13^. To date, many studies have shown that alterations of miRNAs (for example, up-regulation of miR-31, miR-203, miR-146a, miR-155, miR-378a, and down-regulation of miR-99a, miR-125b, miR-194, miR-217, and miR-424) associate with epidermal and dermal changes of psoriatic skin lesions^13–22^. These miRNAs are reportedly demonstrated to participate in regulating different pathways related to the pathogenesis and progression of psoriasis, including inflammation, apoptosis, proliferation, differentiation, and angiogenesis. Recently, a study using laser capture microdissection coupled with high-throughput sequencing showed that miR-378a-3p (herein referred to as miR-378a) was up-regulated specifically in lesional epidermis but not dermis when compared to non-lesional one in psoriatic skin biopsies^13^. In addition to clinical findings, the molecular role of miR-378a has been investigated using in vitro and in vivo models. For example, hypoxic condition and adipogenesis can up-regulated miR-378a level via MAPK1^23,24^. In contrast, down-regulation of miR-378a can induce tumorigenesis and blood vessel formation in mice xenographed with miR-378a-transfected human glioblastoma cell line^25^ MicroRNA-378a also promoted cell viability and suppressed apoptosis in decidual cells via caspase-3 (CASP3)^26,27^. Despite the role of miR-378a in tumorigenesis, its role in keratinocytes during psoriasis development has not been fully elucidated.

Bone morphogenetic proteins (BMPs) are ligand proteins recognized by transforming growth factor-β (TGF-β) superfamily receptors^28^. BMPs are reportedly involved in regulating several pathological processes of skin diseases, including venous ulcers, psoriasis, and skin tumors^29–32^. As one of the BMP sub-family, BMP2 has been reported to inhibit keratinocyte proliferation and promote terminal cell differentiation^33,34^. Additionally, several experiments found that transcription of *BMP2* was decreased in psoriatic skin lesions^35,36^. However, the mechanism underlying the down-regulation of *BMP2* in psoriasis has not been fully clarified.

In this study, we reported that miR-378a expression level was elevated in psoriatic lesions and declined after treatment with MTX or NB-UVB in our patient cohort. We further showed that overexpression of miR-378a was associated with cell cycle arrest of keratinocytes in vitro. Additionally, our transcriptomic analysis of the keratinocytes with miR-378a alterations revealed that *BMP2* was a novel target for miR-378a, supporting the linkage between the presence of miR-378a in keratinocytes and psoriatic pathogenesis.

## Results

### miR-378a expression level elevates in psoriatic skin lesion and declines upon treatment

**Figure 1:**
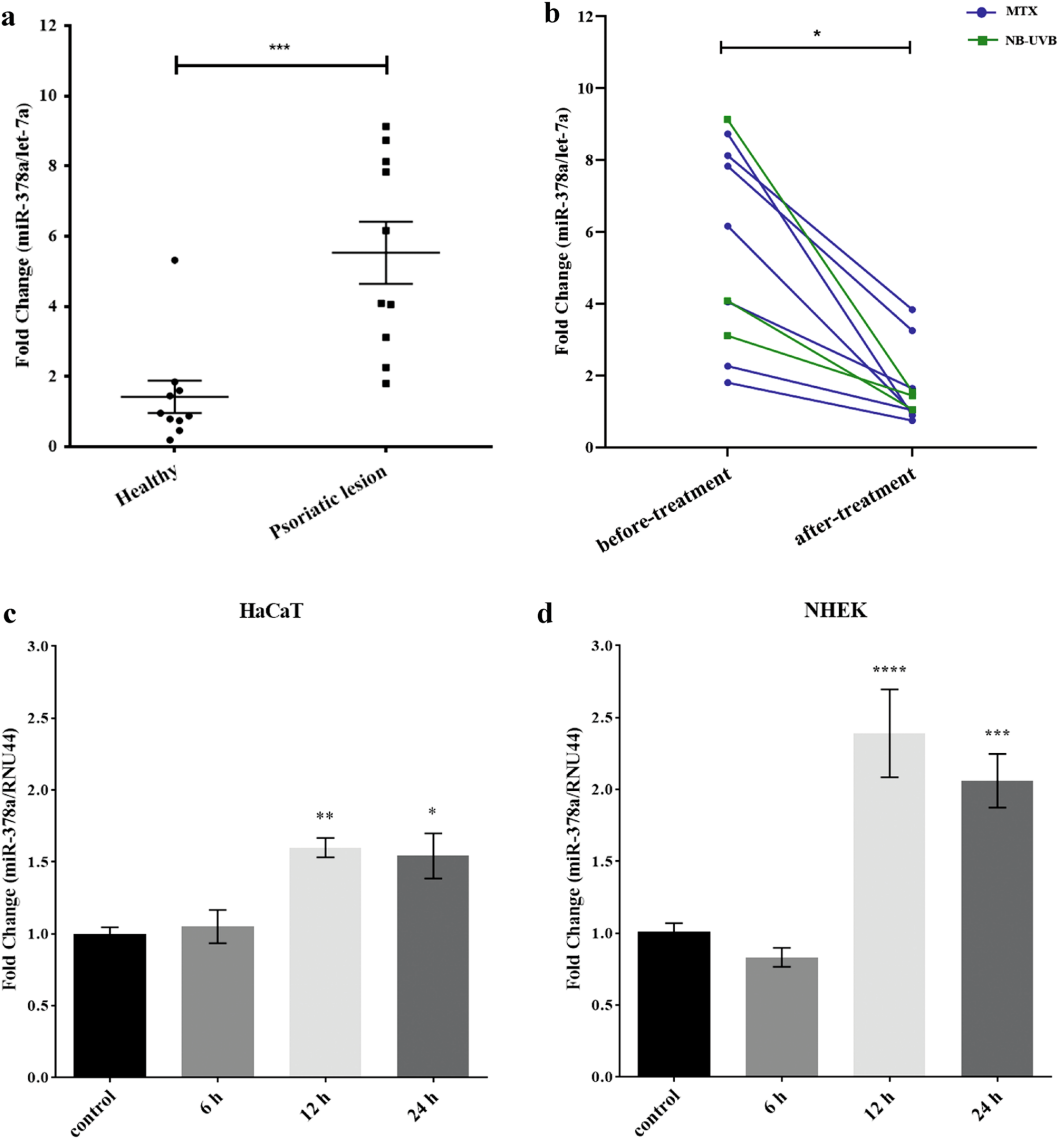
The expression level of miR-378a was elevated in active psoriasis. (**a**) The expression level of miR-378s in psoriatic lesions from Thai patients (n = 10) compared with healthy individuals (n = 10) was analyzed using an unpaired *t-test*. (**b**) The expression level of miR-378a in paired psoriatic skin lesions before and after treatment with MTX (blue; n = 7) and paired psoriatic skin lesions before and after treatment with NB-UVB (green; n = 3) were analyzed using a paired *t-test*. Expression levels of micro RNA were normalized with let-7a expression from the skin biopsies. The expression level of miR-378a in (**c**) immortalized human keratinocytes HaCaT cells, and (**d**) normal human keratinocytes (NHEK) both stimulated with synergistic cytokines (TNF-α + IL-17A) for 6, 12, 24, and 48 h. Expression levels of miRNA were normalized with *RNU44* expression from HaCaT cells. Error bars indicate mean ± SEM, ***indicates *P* < 0.001, **indicates *P* < 0.01, and *indicates *P* < 0.05.

A previous study reported that miR-378a was up-regulated in psoriatic skins^22^, but its expression level in psoriatic patients after treatment of MTX or NB-UVB had not been investigated. To elucidate this, we obtained whole psoriatic skin biopsies from patients and healthy individuals, followed by the measurement of miR-378a expression level by quantitative real-time PCR. Consistent with the previous report, our samples showed a significant increase of miR-378a level compared to healthy individuals (*P* = 0.0003) (Fig. 1a). As expected, the miR-378a expression level in psoriatic skin lesions collected after treatment with MTX (n = 7) or NB-UVB (n = 3) showed a significant reduction of miR-378a level (*P* = 0.0168) (Fig. 1b). However, there was no significant correlation between the miR-378a level and PASI score (data not shown). In general, in vitro stimulation of keratinocyte cell line (HaCaT) with inflammatory cytokines, such as TNF-α and IL-17A, has been used as a model to study the pathogenesis of psoriasis^10^. Moreover, miR-378a expression level was increased and particularly specific in the epidermis of psoriatic skin lesions^13^. From these reports and our previous results (Fig. 1a, b), we hypothesized that miR-378a expression level could be up-regulated after cytokine stimulation in keratinocytes, mimicking psoriatic pathogenesis caused by inflammatory cytokine-producing immune cells. After synergistic stimulation with TNF-α and IL-17A cytokines for 12 h and 24 h, we observed a significant increase of miR-378a expression level in both HaCaT and normal human epidermal keratinocytes (NHEKs) (Fig. 1c, d, respectively), similar to the previous reports by other groups^10,37^. Taken our results together, miR-378a might play a role in psoriatic pathogenesis.

### miR-378a inhibits keratinocyte cell proliferation and increases cell apoptosis

**Figure 2:**
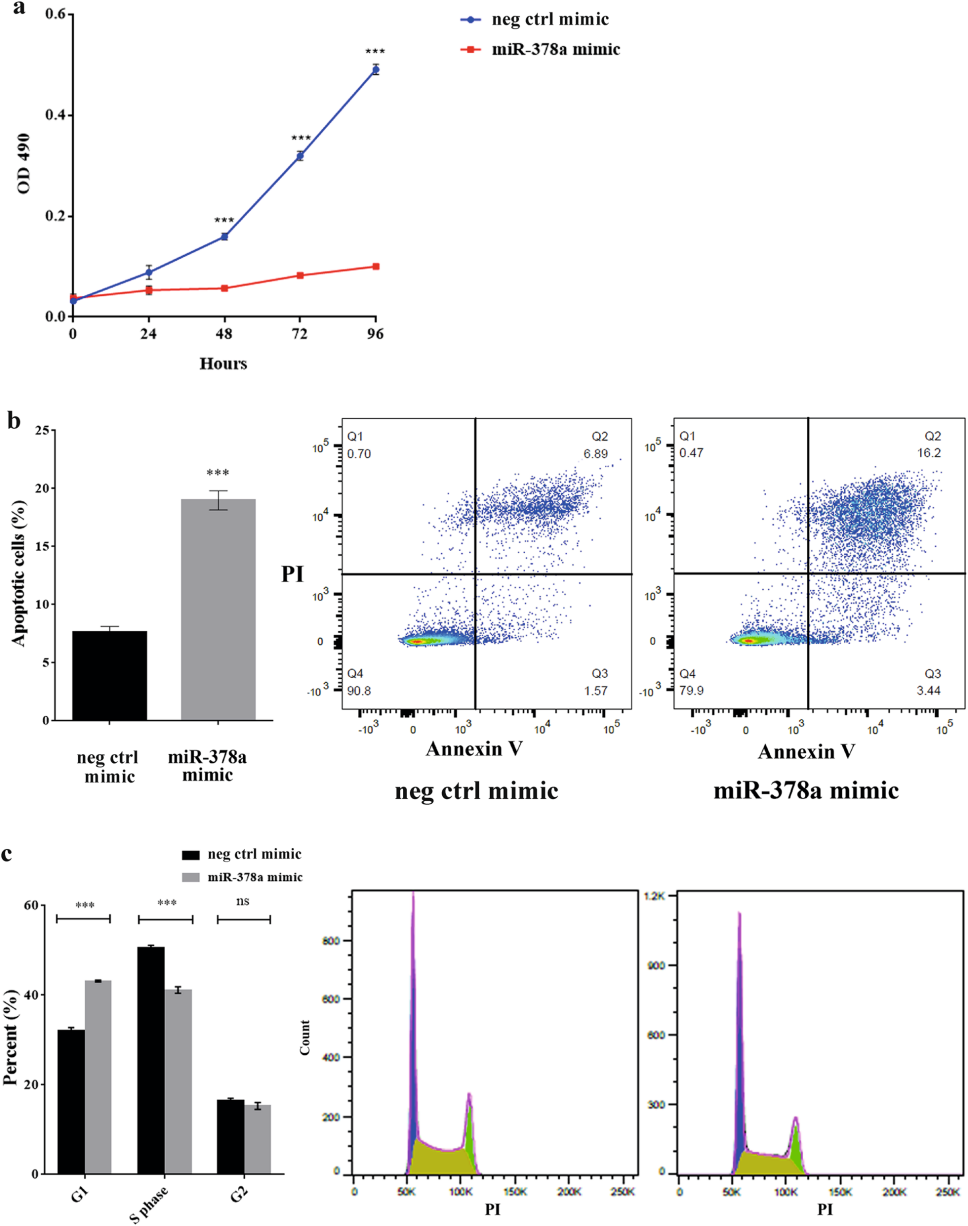
miR-378a diminished cell proliferation in NHEK cells, leading to increased apoptosis and cell cycle arrest. (**a**) The proliferation analysis of NHEK cells transiently expressing miR-378a for 24, 48, 72, and 96 h as measured by MTS assay. (**b**) The percentage of apoptosis keratinocytes after transfection with miR-378a for 72 h. The bar chart shows an average percentage of apoptotic cells calculated from three independent flow cytometry-based apoptosis assays. The dot plots illustrate the necrotic cells (Q1), late apoptotic cells (Q2), early apoptotic cells (Q3), and live cells (Q4). (**c**) The percentage of NHEK cells over-expressing miR-378a for 72 h at different cell cycle phases (G1, S, and G2). The bar chart shows an average of the percentage of cells calculated from three independent cell cycle assays as measured by flow cytometer. The histograms display a representation of cell cycle phase distribution in cells over-expressing miR-378a or negative control mimic. Error bars indicate mean ± SEM from three replicates, ****indicates *P* < 0.0001, ***indicates *P* < 0.001, and ns indicates non-significant. Neg ctrl mimic = negative control of miR-378a.

Because miR-378a was previously reported to play a role in cell proliferation and apoptosis in breast cancer cell line MCF-7^38^, we next hypothesized that miR-378a might regulate keratinocyte proliferation. Using NHEK cells as our in vitro model, we transfected the cells with either miR-378a mimic or miR-378a inhibitor and their negative controls, followed by the measurement of cell proliferation over a 96-h time course. The transfection efficiency is showed in Supplementary Fig. S1a. To our surprise, the proliferation of NHEK cells was diminished when miR-378a was overexpressed (Fig. 2a) whereas the inhibition miR-378a did not affect keratinocyte proliferation (Supplementary Fig. S1b) as measured by a colorimetric MTS assay. Additionally, apoptosis assay also revealed that miR-378a over-expressing cells became more apoptotic as indicated by a higher proportion of dual positive cells (Annexin V^+^ and propidium iodide^+^) when compared to the controls (Fig. 2b). Furthermore, we used propidium iodine staining to examine different cell cycle phases and found that miR-378a overexpression led to cell cycle arrest at the G1 phase at 72 h post-transfection in NHEK cells (Fig. 2c). Taken our results together, the presence of miR-378a led to apoptosis of keratinocytes, possibly through cell cycle arrest at the G1 phase.

### Gene expression profiling of primary human keratinocytes upon overexpression and suppression of miR-378a expression revealed multiple KEGG pathways regulated by miR-378a

**Figure 3:**
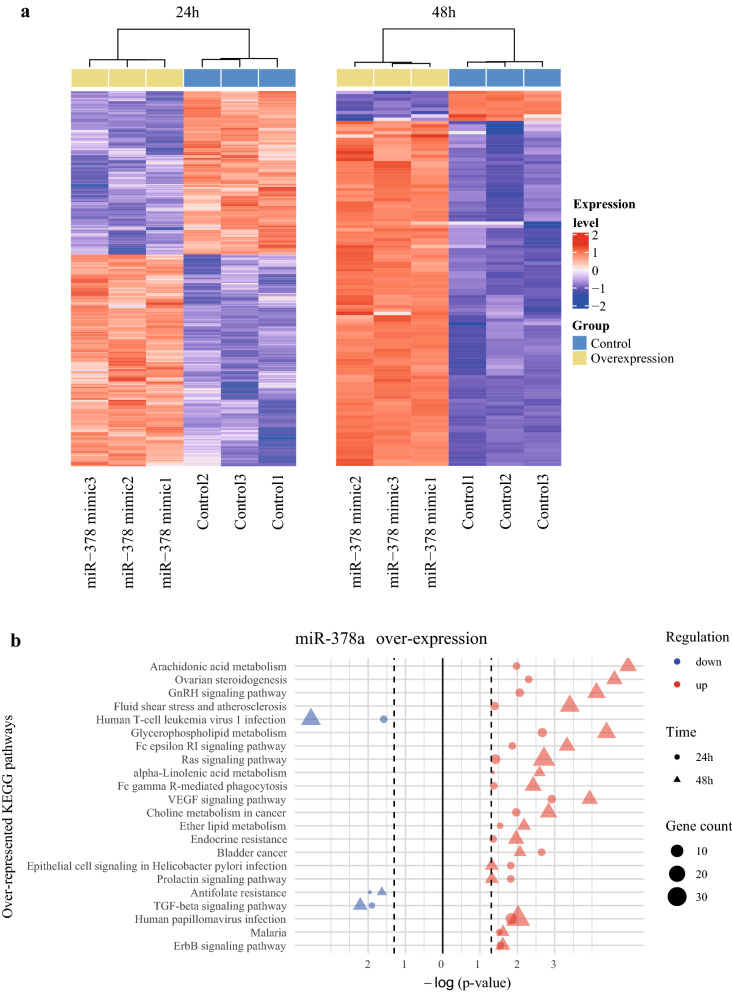
The gene expression profiles in miR-378a overexpressed NHEK cells. (**a**) Heatmap of DEGs in keratinocytes over-expressing miR-378a for 24 h (1233 genes; left) and 48 h (112 genes with |log(fold-change)| > 1.5; right). The heatmaps were drawn using ComplexHeatmap R package. (**b**) The top overlapped KEGG pathways associated with up- and down-regulated genes at both 24 h and 48 h post-transfection of miR-378a mimic. The figures were drawn using tidyverse R package.

**Figure 4:**
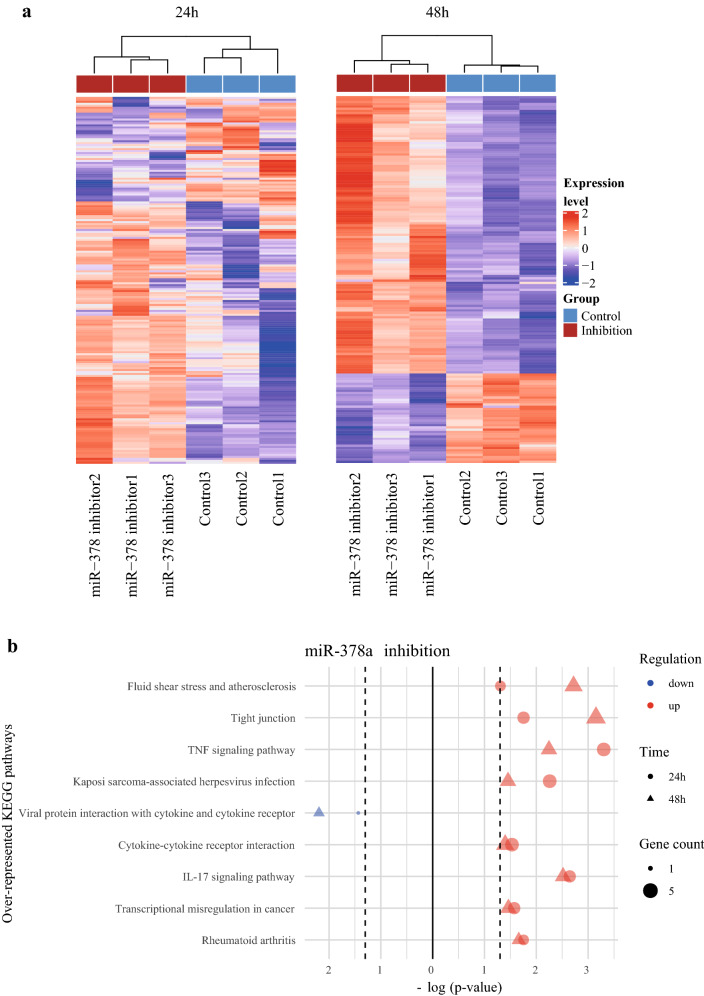
The gene expression profiles in miR-378a suppressed NHEK cells. (**a**) Heatmap of DEGs in keratinocytes with miR-378a suppressing for 24 h (186 genes with |log(fold-change)| > 1; left) and 48 h (160 genes with |log(fold-change)| > 1.5; right). The heatmaps were drawn using ComplexHeatmap R package. (**b**) The top overlapped KEGG pathways associated with up- and down-regulated genes at both 24 h and 48 h post-transfection of miR-378a inhibitor. The figures were drawn using tidyverse R package.

To globally elucidate the downstream pathways affected by miR-378a in keratinocytes, we performed high-throughput RNA sequencing and analyzed the gene expression profiles of the NHEK cells after overexpression or suppression of miR-378a for 24 h and 48 h. We observed that the differentially expressed genes (DEGs) could robustly discriminate the differences between the miR-378a overexpression and the control groups at both time points (Fig. 3a). Besides, the top 25 genes potentially regulated by miR-378a in keratinocytes at each time point were listed in Table 1. To assess the biological function of the DEGs in cells over-expressing miR-378a, we performed an over-representation analysis based on the Kyoto Encyclopedia of Genes and Genomes (KEGG) pathways^39–41^. Using down-regulated DEGs for analysis, we found several pathways were consistently identified at both time points when miR-378a was over-expressed, including TGF-β signaling, human T-cell leukemia virus 1 infection, and antifolate resistance (Fig. 3b). In contrast, we found pathways, such as Fc epsilon RI, Ras, VEGF, and ErbB signaling, to be enriched when using up-regulated DEGs for analysis. A similar analysis was also performed in NHEK cells with miR-378a suppression for 24 h and 48 h. Likewise, the DEGs resulted from miR-378a inhibition could robustly discriminate the differences between the miR-378a inhibition and the control groups (Fig. 4a). Subsequent KEGG pathway analysis of down-regulated DEGs revealed only viral protein interaction with cytokine and cytokine receptor pathway. In contrast, the analysis of up-regulated DEGs enriched tight junction and inflammation-related pathways, such as TNF signaling and IL-17 signaling (Fig. 4b). A complete list of the over-represented KEGG pathways in cells with miR-378a overexpression or suppression at individual time points was also provided in Supplementary Table S1.

**Table 1:** The top (up to 25) down- or up-regulated genes related to miR-378a overexpression or suppression.

miR-378a overexpression				miR-378a suppression			
Down-regulated genes		Up-regulated genes		Down-regulated genes		Up-regulated genes	
24 h	48 h	24 h	48 h	24 h	48 h	24 h	48 h
*SSR3*	*SLC25A6*	*SLC36A1*	*AKAP12*	*ACKR3*	*TUBA4A*	*LUZP6*	*HIST1H1C*
*ELK3*	*NOTCH1*	*SESN3*	*GOLGA7B*	*MIR31HG*	*SYT8*	*ESM1*	*HIST2H2AA3*
*RPN2*	*APLN*	*CXCL14*	*TTC39A*	*NUDT18*	*H19*	*HSPA6*	*LUZP6*
*GALNT3*	*IGFL1*	*FAM110C*	*LY6D*	*CYP26B1*	*CXCL14*	*BOLA2*	*HIST1H2BD*
*SLC25A6*	*UHRF1*	*HMGA2*	*NDRG4*	*GDPD2*	*BCL11A*	*CEACAM6*	*HIST1H2AC*
*RBBP9*	*MSH5*	*PTPRZ1*	*FUT3*	*H19*	*TP73*	*TMPRSS11E*	*IL1RL1*
*ZYX*	*RPN2*	*PTPMT1*	*ABLIM3*	*SNHG26*	*CASP14*	*MUC1*	*HIST2H2BE*
*TMEM250*	*PIM2*	*NTSR1*	*ALOX15B*	*OLFML2A*	*SCN4B*	*ZFP36L1*	*CRCT1*
*TUFT1*	*SNAPC1*	*SNCA*	*GJB6*	*SCN4B*	*LGALS7*	*ATP12A*	*TMEM250*
*FKBP4*	*KIF3B*	*JAG1*	*MMP10*	*TGFBI*	*CYP26B1*	*SLC6A14*	*SSR3*
*UHRF1*	*RAD51AP1*	*EREG*	*COL5A3*		*BMP4*	*FAM25A*	*AKAP12*
*PIM2*	*ZYX*	*MET*	*MEG3*		*KREMEN2*	*CRCT1*	*BOLA2*
*ZDHHC12*	*RBBP9*	*GSE1*	*TNNT1*		*TNFSF10*	*BIRC3*	*MCTP1*
*DAZAP2*	*FKBP4*	*CALU*	*SERPINB1*		*LGALS9B*	*FOXQ1*	*G0S2*
*KIAA1522*	*OAS3*	*ERCC5*	*PKIB*		*IFI44L*	*HEPHL1*	*CSF2*
*CCNK*	*CCK*	*NRBF2*	*MMP1*		*TMEM198B*	*KRT19*	*RNASE7*
*OAS3*	*ZDHHC12*	*TAPT1*	*CASP14*		*CCDC88B*	*GSDMA*	*MMP1*
*CDC23*	*KRT7*	*SLC45A4*	*IL36G*		*OSBPL7*	*PP14571*	*GPAT3*
*IGFL1*	*INPP5D*	*BANF1*	*NTSR1*		*MAPK11*	*SPRR2A*	*FLNC*
*INHBA*	*ELK3*	*ARL4D*	*ACKR3*		*PTGS1*	*IL1R2*	*SPRR2G*
*APLN*	*C4orf46*	*KCNC4*	*PNLIPRP3*		*CRIP2*	*CST6*	*CST6*
*PXDN*	*GALNT3*	*CDK19*	*KCNC4*		*IL33*	*GPRC5A*	*ULBP1*
*TMEM245*	*CCP110*	*SMAP1*	*EPHX3*			*GAB2*	*INHBA*
*ECH1*	*SLC6A9*	*STMN3*	*CEACAM6*			*OXTR*	*HYPK*
*NUAK2*	*KRT13*	*GOLGA7B*	*ATP12A*			*NDRG2*	*LINC-PINT*

### Overexpression of miR-378a results in down-regulation of BMP2 and INHBA

**Figure 5:**
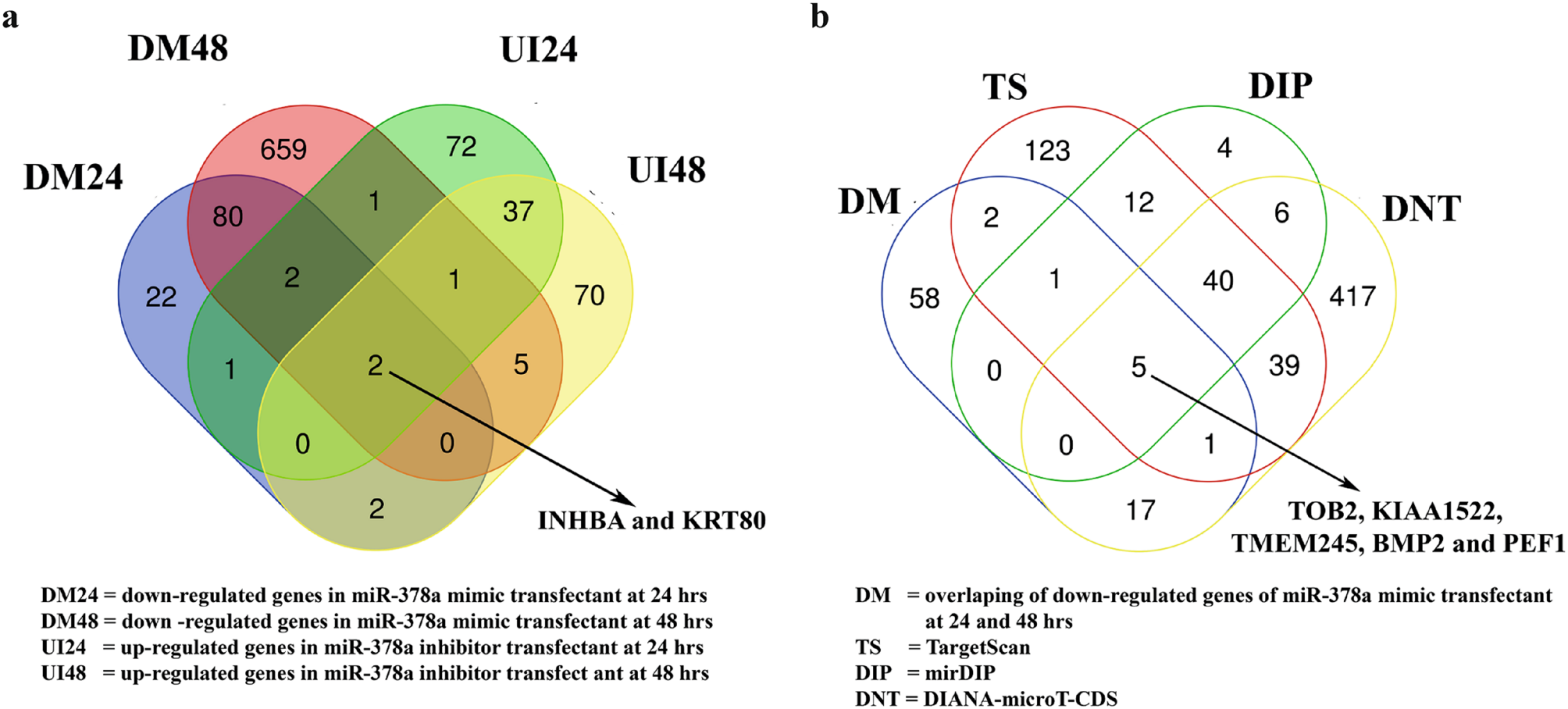
Identification of candidate gene targets of miR-378a. (**a**) Venn diagram shows the overlaps of genes down-regulated (DM) in cells transfected with miR-378a mimic for 24 h and 48 h and genes up-regulated (UI) in cells transfected with miR-378a inhibitor for 24 h and 48 h. (**b**) Venn diagram shows the overlaps of genes identified in (**a**) and target genes of miR-378a predicted by three different databases (see “Materials and methods” section).

**Figure 6:**
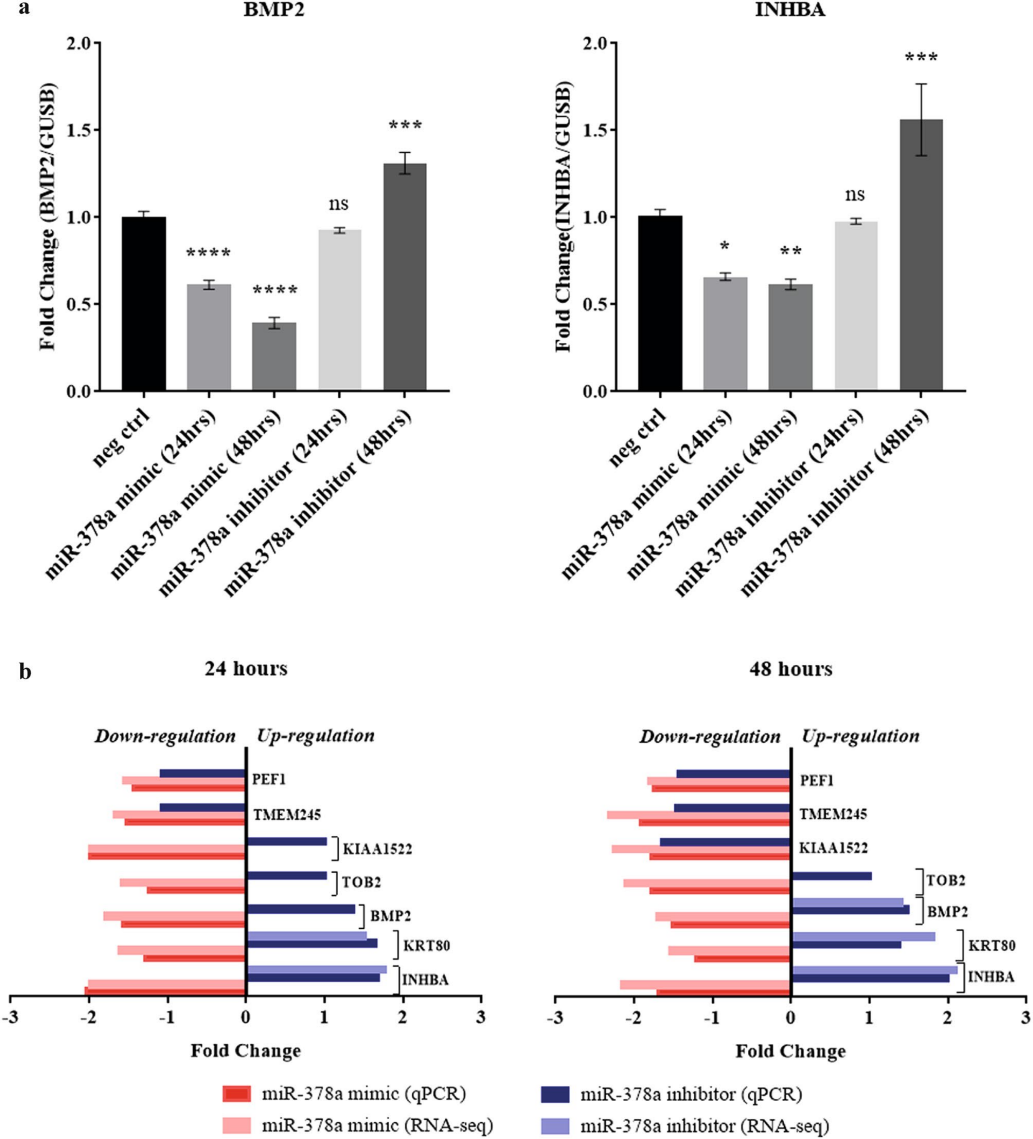
Validation of candidate gene targets of miR-378a. (**a**) The expression levels of *BMP2* and *INHBA* by qPCR using mRNA from NHEK cells transfected with miR-378a mimic or inhibitor for 24 h and 48 h. (**b**) Comparison of the expression levels of the selected candidate genes from high-throughput RNA-sequencing (light colors) versus real-time qPCR (dark colors) from NHEK cells over-expressing miR-378a mimic (red) or inhibitor (blue) for 24 h and 48 h. Error bars indicate mean ± SEM from three replicates. The experiment was performed three times independently. *Indicates *P* < 0.05, **indicates *P* < 0.01, and ***indicates *P* < 0.001.

Having explored the dynamic transcriptomic profiling of keratinocytes in the presence or absence of miR-378a, we next attempted to identify any potential target gene of miR-378a serving as a key regulator of this dynamic change. We hypothesized that the appropriate target gene candidate should be down-regulated in the presence of miR-378a and up-regulated in the absence of miR-378a. By integrating the list of target genes obtained from the gene expression profiles of the keratinocytes from our previous experiments, we identified two overlapping candidate genes: *INHBA* (inhibin beta A) and *KRT80* (keratin 80) (Fig. 5a). Additionally, we searched for more potential target genes of miR-378a from the list of genes predicted as miR-378a targets extracted from three different publicly available databases: 223 candidates from TargetScan 7.2^42^, 525 candidates from DIANA-microT-CDS^43^, and 68 candidates from mirDIP^44^. Combining these candidate genes from different sources, we successfully identified five genes that met our conditions: *BMP2, TOB2* (transducer of ERBB2), *KIAA1522, TMEM245* (transmembrane protein 245), and *PEF1* (penta-EF-hand domain containing 1) (Fig. 5b). They were subjected for validation in NHEK cells in the presence or absence of miR-378a by quantitative mRNA measurement. We found that both *BMP2* and *INHBA* were significantly down-regulated in a time-dependent fashion when miR-378a was overexpressed, and both were significantly up-regulated after 48 h of miR-378a inhibition (Fig. 6a). The results from real-time qPCR were consistent with their expression level from high-throughput RNA-sequencing (Fig. 6b). On the other hand, the patterns of other *BMP2* target genes were disparate (Supplementary Fig. S2). Our results prompted us to further investigate *BMP2* and *INHBA* genes as potential targets of miR-378a.

### BMP2 is directly targeted by miR-378a in human keratinocytes

**Figure 7:**
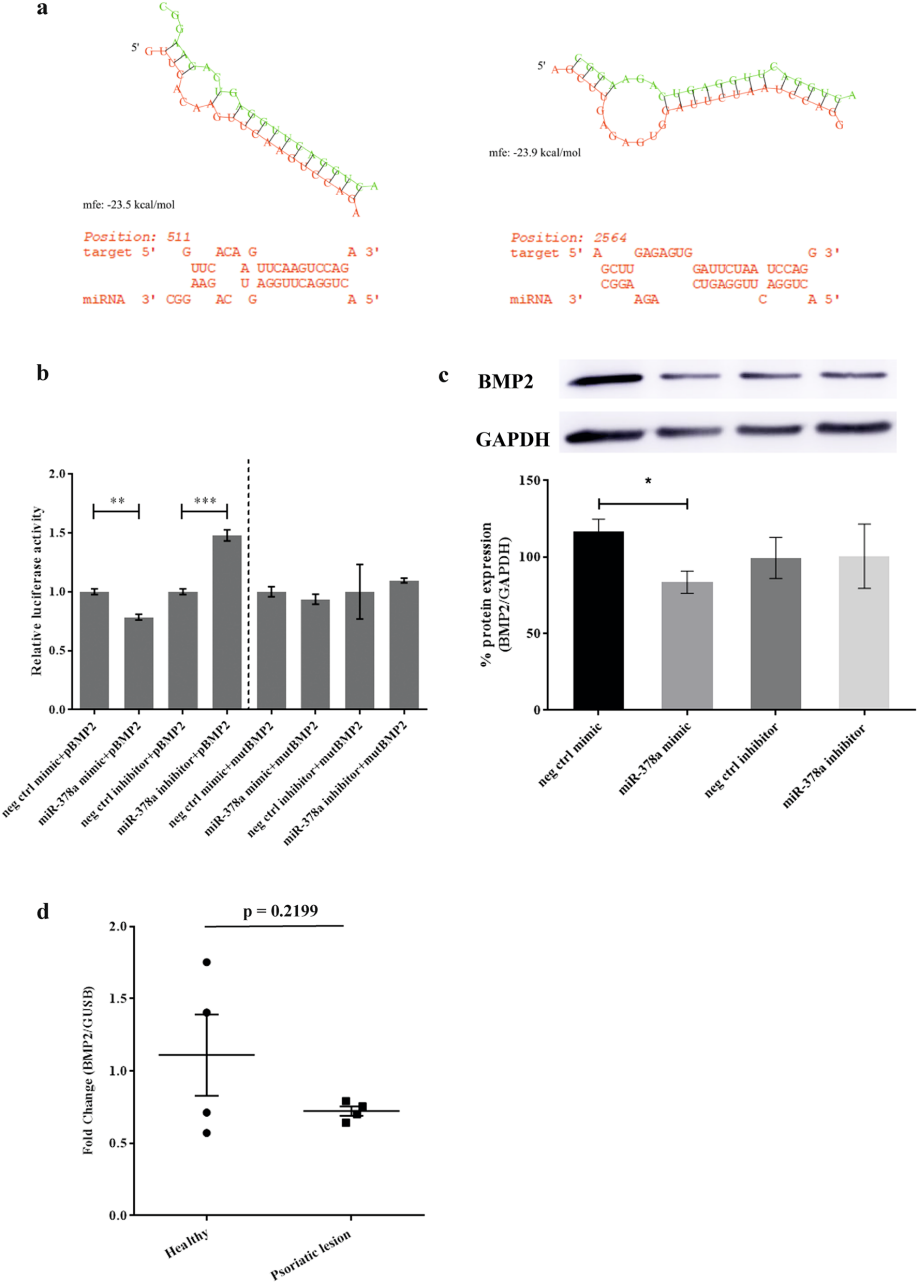
*BMP2* as a novel target gene of miR-378a in keratinocytes. (**a**) Hypothetical binding structure of miR- 378a at 3′UTR of *BMP2* (left) and *INHBA* (right) with predictive binding energy and probability value for actual binding. (**b**) Luciferase activity of wild-type and mutant 3′UTR of *BMP2* genes in HEK293T cells co-transfected with miR-378a mimic or inhibitor for 48 h. (**c**) Western blot images (top) and quantitative measurement (bottom) of BMP2 protein in NHEK cells with miR-378a overexpression or suppression for 72 h. A representative image of BMP2 protein from four independent experiments was shown (also see Supplementary Fig. S5). (**d**) The expression level of *BMP2* in psoriatic lesions (n = 4) and healthy individuals (n = 4). *Indicates *P* < 0.05, **indicates *P* < 0.01, and ***indicates *P* < 0.001.

Having identified *BMP2* and *INHBA* as potential targets of miR-378a, we next asked whether both genes were direct targets of miR-378a. Firstly, we predicted the structure of miR-378a and calculated both binding affinity and free-energy of duplexed RNA structure between miR-378a and 3′UTR of *BMP2* and *INHBA* using RNA hybrid prediction tool^45^. It predicted that miR-378a conserved seed regions could bind to 3′UTR of *BMP2* (mfe = − 23.5 kcal/mol) and *INHBA* (mfe = − 23.9 kcal/mol) (Fig. 7a). To confirm this binding prediction, we used pmiR-Glo dual luciferase plasmid assay containing wild-type (WT) 3′UTR or mutant 3′UTR of either *BMP2* or *INHBA* and co-transfected the plasmid with miR-378a mimic or inhibitor (see Supplementary Table S1 for the complete sequences) into HEK293T cells. As predicted, the luciferase assay showed that miR-378a overexpression significantly reduced the level of WT *BMP2* in contrast to the inhibition of miR-378a that significantly increased the level of WT *BMP2* (Fig. 7b right panel). However, miR-378a did not interact with mutant *BMP2* (Fig. 7b left panel) and both WT and mutant forms of *INHBA* (Supplementary Fig. S3) as indicated by no significant change in the luciferase activity. These results suggested that only *BMP2* was the only direct target of miR-378a. Then, we measured BMP2 protein level in NHEK cells transiently over-expressing or suppressing miR-378a and found that the BMP2 protein level was also significantly reduced at 3 days post-transfection (Fig. 7c). To further confirm our finding in the clinical setting, we measured the expression level of *BMP2* in psoriatic lesions from our cohort and found that biopsies from psoriasis patients had substantially lower *BMP2* expression level, but not significantly, when compared to the healthy individuals (Fig. 7d, *P* value = 0.2199). Taken together, our results suggested that miR-378a could directly bind to *BMP2* and subsequently decreased its expression level in keratinocytes in vitro.

## Discussion

Keratinocytes are the most abundant cell type in the epidermis and play an important role in induction, maintenance, and amplification of the chronic phase of psoriatic disease^4,5^. Alteration of miR-378a expression in keratinocytes emphasizes the critical role of miR-378a in the pathogenesis of psoriasis. Previous studies reported the high expression level of miR-378a in the biopsies, epidermis and keratinocytes of the psoriatic lesion^13,22,46^. Similarly, we also found that miR-378a was up-regulated in active psoriatic skin lesions and down-regulated upon treatment with two conventional therapies (MTX and NB-UVB). Besides, we also demonstrated that the expression of miR-378a was significantly increased in keratinocytes synergistically stimulated with TNF-α and IL-17A in vitro, consistent with the recent report by Pasquali and colleagues^47^.

To underline the cellular processes regulated by miR-378a in keratinocytes, we assessed cell proliferation and apoptosis. To our knowledge, we were the first to report that miR-378a diminished proliferation, increased apoptosis and identified BMP2 as a direct target of miR-378a in keratinocyte cells. Additionally, the high-throughput RNA-sequencing revealed that overexpression of miR-378a in keratinocyte in vitro model increased pro-apoptotic genes, such as *TNFSF10* and *TNFSF15* (data not shown), confirming that the miR-378a indeed induced apoptosis. Previously, a study in gamma-irradiated primary human epidermal keratinocytes reported that attenuated G1 arrest correlated with less p53 tumor suppressor^48^. In colon cancer, p53 expression level was enhanced by inhibition of Gli3 transcription factor using siRNA^49^ whereas Gli3 has been reported as of the miR-378a targets in liver fibrosis^50^. Because we found that TOB2, another miR-378a target, was significantly reduced in miR-378a expressing keratinocytes (Supplementary Fig. S2) in addition to its ability to suppress cyclin D1 that controls G1 exit^51^, the increased G1 cell cycle arrest observed in our model may be regulated through miR378-a/Gli3/p53 axis independently of cyclin D1. However, this hypothesis is pending further investigation.

Although our samples came from whole psoriatic skin biopsies, other studies also examined gene expression profiles of psoriatic lesions and non-lesions. They reported that miR-378a was indeed highly expressed in the epidermis layer of psoriatic lesions when compared to other layers^13,46^. In addition to the study reporting apoptotic keratinocytes abundantly found in stratum corneum from psoriatic lesions^52^, another study showed that apoptosis could also be induced via interaction of the soluble TNF-like weak inducer of apoptosis (TWEAK or TNFSF12) and membrane-bound fibroblast growth factor-inducible 14 receptor (FN14 or TNFRSF12A) in keratinocyte in vitro models, such as HaCaT and NHEK cells, as well as in psoriatic lesions^53^. However, whether this TWEAK/FN14 axis also plays a regulatory role in miR-378a-induced apoptosis in keratinocytes requires further investigation.

Our KEGG pathway analysis revealed an association between miR-378a and various pathways, possibly underlining cellular processes of keratinocytes. TGF-β signaling pathway was among the significant pathways identified from the set of down-regulated DEGs in miR-378a-ooverexpressed keratinocytes. Down-regulation of TGF-β and several TGF-β-mediated signaling molecules were observed and regulated cell proliferation in psoriatic lesions^54–56^. Similar observation was also observed in hepatic stallate cells and liver fibrosis wherein miR-378a was overexpressed^50^. Taken together, our transcriptomic results suggest that miR-378a involved in regulation of TGF-β signaling pathway in psoriatic keratinocytes.

Tight junction proteins are also essential not only in regulation of cell permeability but also in enrolling different signaling proteins involving in cell proliferation and differentiation regulations^57^. The observed tight junction pathway significantly enriched in miR-378a-deficient keratinocytes was consistent with the previous studies reporting that down-regulation of tight junction proteins (i.e., claudin‐1, -3, -7, and zonula occludens 1) were correlated with an increase of interleukin-36γ (IL-36γ) and a decrease of vitamin D receptor (VDR) expression in the epidermis of psoriatic lesions^58–62^. From this observation, it suggests that miR-378a may promote the dysregulation of tight junction proteins, exacerbating the disease pathogenesis. However, an investigation on the role of miR-378a on the tight junctions in the psoriatic epidermis is probably required.

It is widely known that BMP2 inhibits primary keratinocyte proliferation via SMAD1/5/8, resulting in promoting keratinocyte differentiation, proliferation, and inhibition of apoptosis^33,63^. However, we presented that BMP2 was a direct target of miR-378a, according to the data from our luciferase reporter assay and protein detection. We also found that keratinocyte differentiation marker, involucrin, was down-regulated but not significantly (Supplementary Fig. S4). Despite this discrepancy, our results were in line with one study in ovarian cancer stem-like cells (CSCs), demonstrating that BMP2 was preferentially expressed in different CSC populations. In particular, BMP2 promotes cell expansion of aldehyde dehydrogenase (ALDH) and CD133 dual-positive population, which are highly tumorigenic^64^. Moreover, in myoblast cells, miR-378a expression was found to target histone deacetylase 4 (*HDAC4*), and therefore it inhibited proliferation, promote apoptosis and differentiation of the cells^26^.

Another non-direct target of miR-378a is *INHBA* (inhibin beta A) encodes for proβAβA protein^65^. We showed that *INHBA* was significantly down-regulated in the presence of miR-378a in NHEK cells (Fig. 6a, b). Still, the luciferase activity revealed that the *INHBA* was not directly targeted by miR-378a, suggesting that *INHBA* was not a direct target of miR-378a. Recently, *INHBA* can be induced by BMP2 through ALK3BMP2R/ACVR2A-SMAD1/5/8-SMAD4 axis in human extravillous trophoblast cells^66^. Taken together, *INHBA* may be indirectly regulated miR-378a through BMP2 and its downstream ALK3BMP2R/ACVR2A-SMAD1/5/8-SMAD4 pathway. Additionally, miR-378a was recently demonstrated to target *PPARα* for degradation, reducing oxidative stress activity in hepatocytes^67^. Because oxidative stress can induce proliferation of psoriatic lesions, we speculate that in keratinocytes the miR-378a may target *PPARα* for mRNA degradation and subsequently down-regulate oxidative stress, resulting in diminished proliferation. However, more investigation is needed to elucidate whether miR-378a induces apoptosis through either ALK3BMP2R/ACVR2A-SMAD1/5/8-SMAD4 pathway or *PPARα* degradation in keratinocyte in vitro model and psoriatic lesions.

In conclusion, the present study has shown that miR-378a expression was found in active psoriatic skins and inflammatory cytokine-stimulated keratinocytes. The high level of miR-378a attenuated keratinocyte proliferation by causing cell cycle arrest and negatively regulated inflammation- and tight junction-related pathways. miR-378a directly targeted *BMP2*, an essential element activating TGF-β signaling cascade, which has been shown to be diminished in the presence of miR-378a. Our findings highlight the regulatory role of miR-378a in the pathogenesis of psoriasis and prove that it directly binds to *BMP2* gene, offering a novel target for miR-378a-mediated psoriasis. However, other target genes of miR-378a should be further validated to confirm the function in cellular processes.

## Materials and methods

### Collection of skin biopsies

The study protocol was approved by the Institutional Review Board at the Faculty of Medicine, Chulalongkorn University (IRB number 516/57) and performed under the guidelines and regulations mandated by the board. Written informed consents were obtained from psoriasis patients and healthy individuals before enrollment in the study. Skin biopsies from 10 patients diagnosed with moderate to severe chronic plaque-type psoriasis at King Chulalongkorn Memorial Hospital (Bangkok, Thailand) and ten normal subjects were analyzed in this study. The severity of psoriasis was classified according to the Psoriasis Area and Severity Index^68^ (PASI score ≥ 10 = moderate to severe). Seven patients were orally treated with 15 mg MTX once a week, and three patients were treated with NB-UVB irradiation three sessions per week for up to 12 weeks as a monotherapy. Skin biopsies from patients with psoriasis were obtained before and after 4-week monotherapy with either MTX or NB-UVB. Normal skin tissue biopsies were collected from healthy subjects who underwent plastic surgery. Patients with psoriatic arthritis, other autoimmune diseases, cancer, liver or renal disease were excluded from the study. All patients were free from all systemic therapies and photo-therapies for at least four weeks and topical anti-psoriatic treatments for at least two weeks before specimen collection.

### MicroRNA expression in skin samples

Unless otherwise specified, all reagents were purchased from Thermo Fisher Scientific. The level of miR-378a expression was determined by quantitative real-time PCR (qPCR). Firstly, the enriched microRNAs (< 200 bp) were extracted from whole-biopsies using the miRNeasy Mini Kit (#217004, Qiagen) according to the manufacturer’s instructions for microRNA enrichment protocol. Then, 10 ng of enriched microRNAs was reverse-transcribed in 15 μl reaction with the Applied Biosystems TaqMan MicroRNA Reverse Transcription Kit (#4366596), and 1.33 μl of cDNA was added to triplicate in 20 μl PCR reactions. The qPCR reaction was performed on Applied Biosystems 7900HT thermocycler using Applied Biosystems TaqMan Universal PCR Master Mix (#4305719). For amplification of specific microRNA, hsa-miR-378a-3p (ID#001314) and hsa-let-7a (ID#000377) probe-primer set were applied following the manufacturer’s instructions. The level of miR-378a expression was normalized to the let-7a, and relative expression levels were calculated according to the 2^−∆∆Ct^ method^69^.

### MicroRNA expression in keratinocytes

Normal human epidermal keratinocyte cells (NHEK; ATCC PCS-200-010) were purchased from ATCC. NHEKs were maintained in Dermal Cell Basal Medium supplemented with keratinocyte growth kit (ATCC PCS-200-040; 0.4% bovine pituitary extract (BPE), 0.5 ng/ml rh TGF-α, 6 mM l-glutamine, 100 ng/ml hydrocortisone hemisuccinate, 5 mg/ml recombinant human insulin, 1.0 mM epinephrine, 5 mg/ml apo-transferrin, 33 µM phenol red and penicillin–streptomycin–amphotericin B solution) and were incubated at 37 °C in a humidified incubator containing 5% CO_2_. Additionally, HaCaT cells (RRID:CVCL_0038) were maintained in Dulbecco’s Modified Eagle Medium (DMEM, high glucose, pyruvate; #11995) and supplemented with 10% fetal bovine serum (#10270), 0.01 M HEPES (#15630) and 100 U/ml penicillin–streptomycin (#15140). Cells were incubated at 37 °C in a humidified incubator containing 5% CO_2_. Both cells in each well were treated with combinations of human recombinant cytokines: 10 ng TNF-α and 200 ng IL-17A (#210-TA and #317-ILB, respectively, R&D Systems Inc.), for 6, 12 or 24 h before collecting for miRNA evaluation.

### Cell culture and transient transfection of miRNA plasmids

NHEKs (passage 3) were seeded at 5 × 10^4^ cells per well in 12-well plates for 6 h and then were transiently transfected with 100 nM hsa-miR-378a-3p mimic (#MC10049), negative control mimic (#4464058), 100 nM hsa-miR-378a-3p inhibitor (#MH11360) or negative control inhibitor (#4464076). All were purchased from Invitrogen mirVana miRNA (Thermo Fisher Scientific). Transient transfections were performed using Invitrogen Lipofectamine RNAiMax Transfection Reagent (#13778075) according to the manufacturer’s instructions. We observed a significantly high expression level of miR-378a at 24, 48 and 72 h post-transfection, whereas miR-378a inhibitor significantly decreased miR-378a expression level at 48 h post-transfection (Supplementary Fig. S1).

### Cell proliferation assay

The transfected NHEKs were trypsinized and seeded at 3 × 10^3^ of a 96-well plate. Cell proliferation was evaluated with CellTiter 96 AQ_ueous_ One Solution Cell Proliferation Assay according to the manufacturer’s instruction (#G3582, Promega) at 0, 24, 48, 72, and 96 h. Briefly, at each time point, 20 μl of MTS solution was added to 100 μl of culture medium containing transfected cells and incubated for three hours at 37 °C in a 5% CO_2_ incubator. The absorbance was evaluated at OD 490 nm using Thermo Scientific Varioskan Flash Multimode Reader. The experiments were independently performed in triplicates.

### Cell apoptosis and cell cycle assays

NHEK was transiently transfected for 48 h and then replaced with a new medium for 24 h. The apoptosis assay was performed with flow cytometry using FITC Annexin V Apoptosis Detection Kit with PI (#640914, BioLegend) according to the manufacturer’s protocol. Briefly, all cells were trypsinized and washed with DPBS buffer (ATCC 30-2200) twice. Afterward, the cells were stained with FITC Annexin V and PI for 15 min in the dark at room temperature. Besides, the apoptotic cells were determined by BD LSR II flow cytometer (BD Biosciences) and evaluated by FlowJo v10 software (FlowJo LLC). Moreover, the transfected cells were fixed by adding drop-size cold 70% ethanol for four hours at 4 °C to measure cycle assay. Afterward, the fixed cells were washed with DPBS buffer twice and treated with 50 µl of a 100 µg/ml RNaseA. Then, the cells were stained with 200 µl of a 50 µg/ml PI (#42130, BioLegend). After staining, the cells were measured by BD LSR II flow cytometer and analyzed using FlowJo v10 software for cell cycle analysis. The experiments were independently performed in triplicates.

### RNA library preparation and sequencing

Transiently transfected cells were collected at 24 and 48 h after transfection. Total RNA was extracted using Ambion TRIzol Reagent (#66115, Sigma-Aldrich) according to the manufacturer’s instruction. The next-generation sequencing was performed by a commercial sequencing facility (Macrogen, Seoul, Korea). Briefly, RNA quality and quantity were checked using an Agilent 2100 Bioanalyzer with an RNA Integrity Number (RIN). Libraries for high throughput sequencing were prepared using the TruSeq Stranded mRNA LT Sample Prep Kit. The cDNAs were checked for quality and quantity on an Agilent 2100 Bioanalyzer. RNA library sequencing was carried on using Illumina NovaSeq 6000 System with 100 bp paired-end reads. The quality control of the sequenced raw reads was analyzed. In order to reduce biases in the analysis, low-quality reads, adaptor sequence, contaminant DNA, or PCR duplicates were removed. Trimmed reads were mapped to a reference genome (UCSC hg19) with HISAT2^70^. Transcripts were assembled by StringTie^71^ with aligned reads. Gene differential expression analysis was performed with edgeR^72–74^ in R version 4.0.1^75^ and RStudio^76^. The cut-off criteria (*P* value < 0.01 and FDR < 0.05) were applied to select differentially expressed genes. The overlapping genes were identified and Venn diagrams were drawn using an online tool (http://bioinformatics.psb.ugent.be/webtools/Venn/). The sequencing data in this publication are available in the National Center for Biotechnology Information’s Gene Expression Omnibus (NCBI GEO database)^77^, and they are accessible through GEO Series accession number GSE160906 (https://www.ncbi.nlm.nih.gov/geo/query/acc.cgi?acc=GSE160906).

### Quantitative real-time PCR (qPCR) and pathway analysis of transcriptome profiles

cDNA samples were synthesized using Applied Biosystems High-Capacity cDNA Reverse Transcription Kit according to the manufacturer’s instruction. qPCR reactions were carried out in 10 μl consisting of 500 ng of cDNA in duplicate. qPCR was performed on Applied Biosystems 7900HT thermocycler. The list of primers used for the detection of genes of interest in this work was provided in Supplementary Table S2. The mRNA expression was normalized with the expression of the housekeeping gene *GUSB*^78^. Relative expression levels were calculated according to the 2^−∆∆Ct^ method^69^. Normalized expression levels of the DEGs with FDR < 0.05 were displayed in the heatmaps. For transcriptome profile analysis, we performed Gene Ontology^79,80^ and KEGG^39–41^ enrichment analysis using the online tool Enrichr^81–84^. The results were depicted using clusterProfiler^85^, DOSE^86^, ComplexHeatmap^87^ and tidyverse^88^ R packages.

### 3′UTR-luciferase reporter analysis

The miR-378a binding sites and binding efficiency in the presence of the 3′UTR of miR-378a-predicted targets (*BMP2* and *INHBA*) were predicted by RNAhybrid^45^ (with a minimum free energy (mfe) threshold of − 20 to − 30). Then, these binding sites were constructed and ligated into pmirGLO Dual-Luciferase miRNA Target Expression Vector (#E1330, Promega). One microgram of plasmid was co-transfected with the miR-378a mimic/negative control or inhibitor/negative control to HEK293T (ATCC CRL-3216). After 48 h, the samples were collected to be measured using the Dual-Luciferase Reporter Assay System (#E1910, Promega) according to the manufacturer’s protocol.

### Western blotting and analysis

NHEK cells were seeded and then transfected with miR-378a mimic or inhibitor and its negative control, as described previously. To collect protein lysate for Western blot analysis, at 48 h post-transfection, we trypsinized cells and washed with DPBS buffer twice. Then, the cells were lysed with Millipore RIPA Lysis Buffer (#20-188, Merck) containing Thermo Scientific Halt Protease Inhibitor Cocktail (#78430). The protein concentration was quantified using Pierce BCA Protein Assay Kit (#23225). To detect the protein expression of BMP2, involucrin, and GAPDH, 20 μg of protein from the same experiment were separately loaded into the SDS-PAGE gels and processed in parallels. Briefly, ten microliters of protein marker (Precision Plus Dual Color Standards; #1610374, Bio-Rad Laboratories Inc.) and 20 μg of protein from each sample were separately loaded into 10% SDS-PAGE gels, run at 100 V for 60 min, and transferred onto a polyvinylidene difluoride membrane (#10600023, Amersham Hybond P 0.45 um PVDF membrane, Cytiva Life Sciences). The membranes were cut according to molecular weight of the proteins and initially blocked with blocking buffer (5% non-fat dry milk in PBST) for 1 h. Later, it was immunoblotted with anti-BMP2 antibody (1:1000 dilution; #AHP2442, Bio-Rad Laboratories, Inc.), anti-involucrin antibody (1:1000 dilution; #sc-398952, Santa Cruz Biotechnology), and anti-GAPDH (1:5000 dilution; Santa Cruz Biotechnology, Dallas, TX, USA; cat#sc-25778) for 1 h at room temperature. After washing three times with washing buffer, the membranes were incubated with goat anti-rabbit(H + L)-HRP (1:5000 dilution; #1706515, Bio-Rad Laboratories Inc.) or goat anti-mouse IgG-HRP (Cruz Marker) (1:5000 dilution; #sc-2031, Santa Cruz Biotechnology) for 1 h at room temperature. The protein signals were enhanced using Thermo Scientific SuperSignal West Femto Maximum Sensitivity Substrate (#34094) and digitally recorded using an ImafeQuant (Cytiva Life Sciences). Image Studio Lite software version 5.2 (LI-COR Biosciences) was used to analyze the protein signals quantitatively.

### Statistical analysis

All data were represented as mean ± standard error of the mean (SEM) based on at least three independent experiments. A two-tailed Student’s t-test was used to determine the significant difference between the two groups and the data with more than two groups were determined by one-way ANOVA followed by Turkey’s post-hoc testing. GraphPad Prism version 6 (GraphPad Software Inc.) was used to perform statistical analysis. *P* values < 0.05 were considered significant differences among means.

## Supplementary information

Supplementary Information.
