# Supplementary material for: MicroRNA-378a-3p is overexpressed in psoriasis and modulates cell cycle arrest in keratinocytes via targeting BMP2 gene
Source: Sci Rep. 2021 Jul 9;11:14186. doi: 10.1038/s41598-021-93616-8 (PMC8270917; doi:10.1038/s41598-021-93616-8)
Supplement: Supplementary file 1 — Supplementary Information. [file 41598_2021_93616_MOESM1_ESM.pdf]

Supplementary Information

MicroRNA-378a-3p is overexpressed in psoriasis and modulates cell cycle arrest in keratinocytes via targeting BMP2 gene

Wipasiri Soonthornchai[1], Pattarin Tangtanatakul[1,2], Patipark Kueanjinda[1], Kornvalee Meesilpavikkai[3], Virgil Dalm[4], Jongkonnee Wongpiyabovorn[1,*]

[1]Center of Excellence in Immunology and Immune-Mediated Diseases, Department of Microbiology, Faculty of Medicine, Chulalongkorn University, Bangkok, Thailand 10330

[2]Department of Transfusion Medicine and Clinical Microbiology, Faculty of Allied Health Sciences, Chulalongkorn University, Bangkok, Thailand 10330

[3]Division of Mycology, Department of Microbiology, Faculty of Medicine, Chulalongkorn University, Bangkok, Thailand 10330

[4]Division of Clinical Immunology, Department of Internal Medicine, Erasmus University Medical Center, Rotterdam, The Netherlands

Supplementary Figure S1. Transfection efficiency of miR-378a mimic and inhibitors in NHEK cells. **a**, Percentage of miR-378a remaining after NHEK cells were transfected with miR-378a mimics or inhibitors for 24 hours, 48 hours and 72 hours. **b**, The proliferation analysis of NHEK cells transiently expressing miR-378a inhibitor for 24, 48, 72, and 96 hours as measured by MTS assay. Error bars indicate mean ± SEM, **** indicates $P < 0.0001$, *** indicates $P < 0.001$, and ns indicates non-significant.

Supplementary Figure S2. Expression levels of candidate genes. Genes identified in Figure 5 (*KIAA1522, KRT80, PEF1, TMEM245, TOB2*) were measured by qPCR using mRNA from NHEK cells transfected with miR-378a mimic or inhibitor for 24 hours and 48 hours. Error bars indicate mean ± SEM from 3 replicates. The experiment was performed three times independently. * indicates $P< 0.05$, ** indicates $P< 0.01$, and *** indicates $P< 0.001$.

Supplementary Figure S3. Validation of miR-378a binding to *INHBA*. Luciferase activity of wild type (pINHBA) and mutant 3'UTR of *INHBA* genes (mutINHBA) co-transfected with miR-378a mimic or inhibitor and their negative control in HEK293T cells for 48 hours. Error bars indicate mean ± SEM, **** indicates $P < 0.0001$, and *** indicates $P < 0.001$.

Supplementary Figure S4. The effect of miR-378a on keratinocyte differentation marker involucrin. Western blotting was used to detect protein expression levels of involucrin in NHEK cells over-expressing miR-378a mimic or inhibitor and their negative coltrol for 72 hours. One representative blot from four independent experiments (see Supplementary Fig. S5) was shown (top). Relative expression levels from densitometric analysis of the four blots was depicted as bar chart (bottom). Error bars indicate mean ± SEM, **** indicates $P < 0.0001$, and *** indicates $P < 0.001$.

Supplementary Figure S5. **Raw images of Western blots**. Western blots were used to detect BMP2, involucrin, and GAPDH proteins in NHEK cells overexpressing miR-378a mimic or inhibitor and their negative control for 72 hours (3 days). Four independent experiments were performed (a-d).

Table legends

Supplementary Table S1. Results of KEGG pathway analysis. KEGG pathway analysis by Enrichr using (**a**) DEGs from NHEK cells transfected with miR-378a mimic or its control for 24 and 48 hours and (**b**) DEGs from NHEK cells transfected with miR-378a inhibitor or its control for 24 and 48 hours.

Supplementary Table S2. List of primers used for real-time PCR in this study.

Supplementary Table S3. List of miR-378a, *BMP2* 3' UTR, *BMP2* 3' UTR mutant, *INHBA* 3' UTR and *INHBA* 3' UTR mutant used in the luciferase study.

a

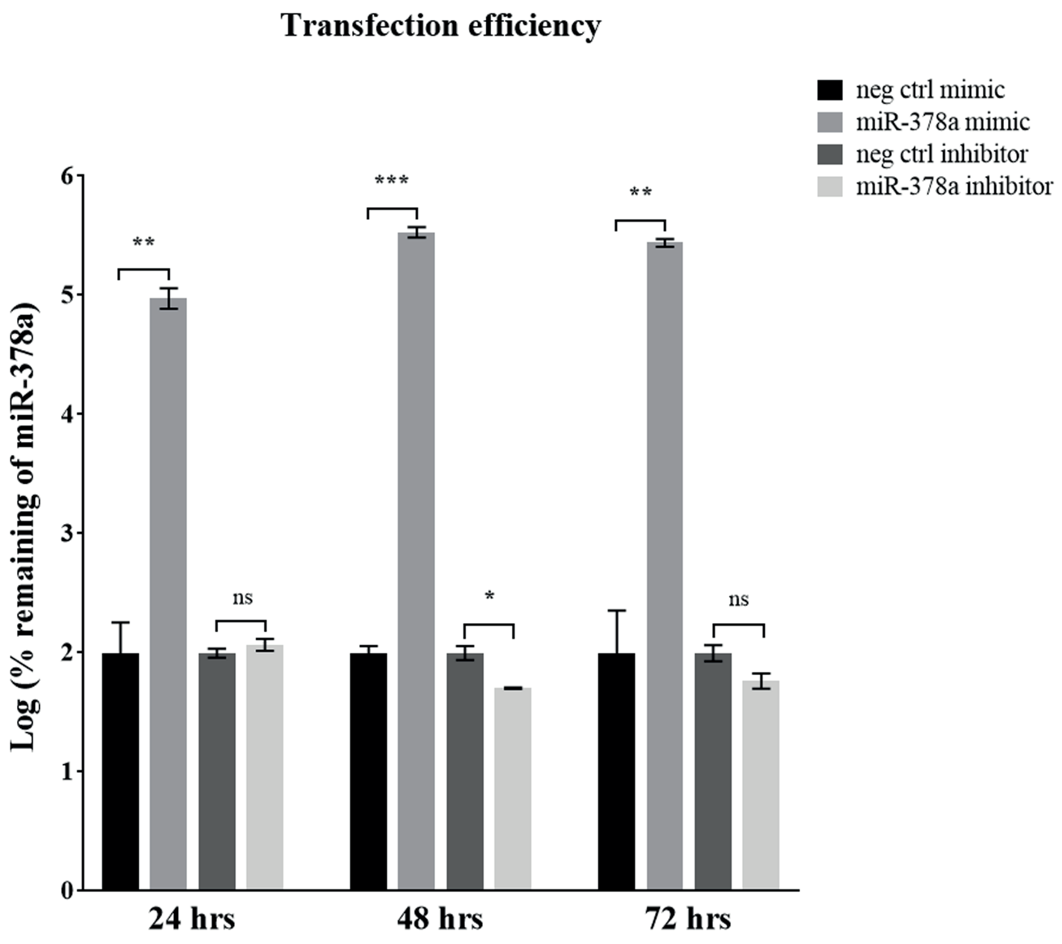

b

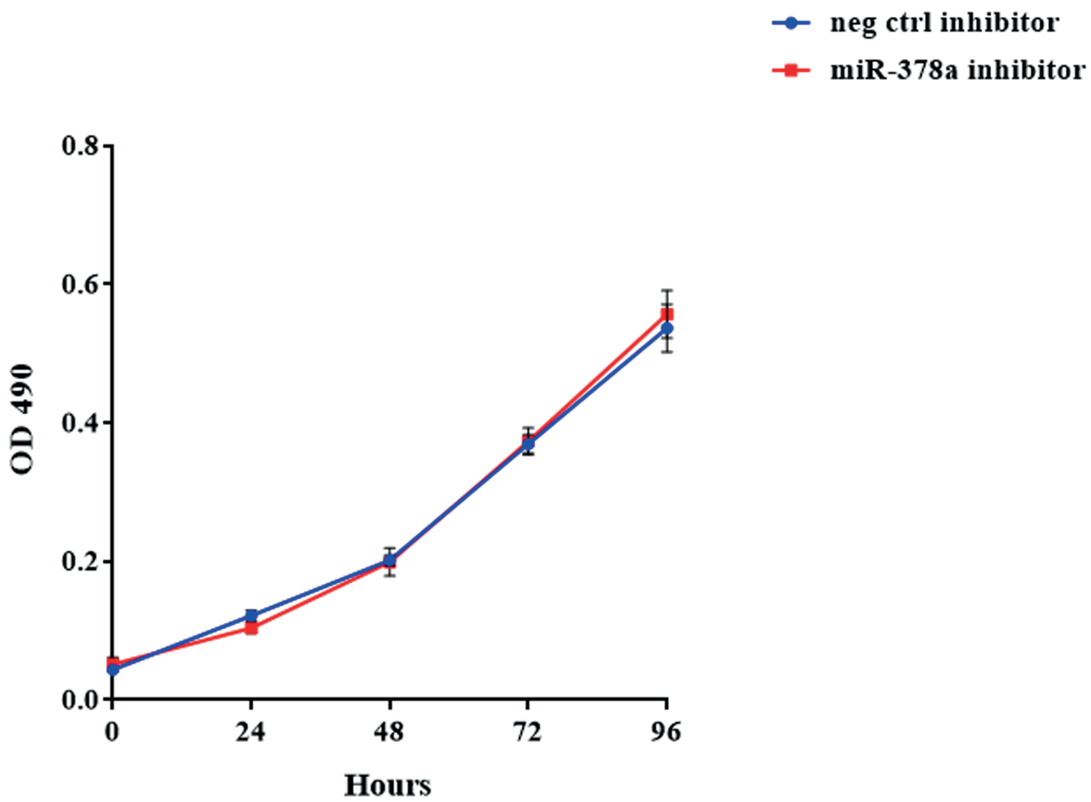

Supplementary Figure S1

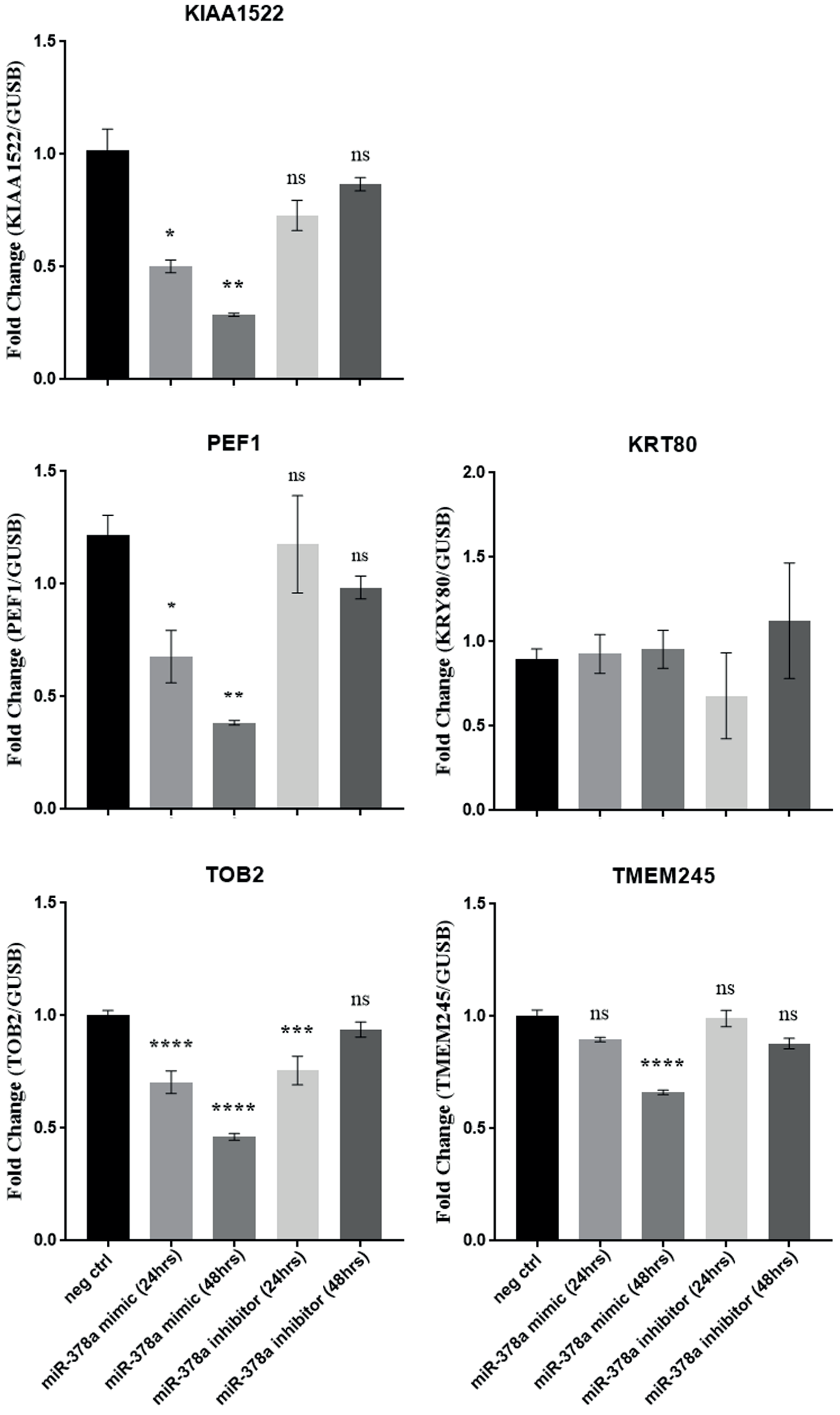

Supplementary Figure S2

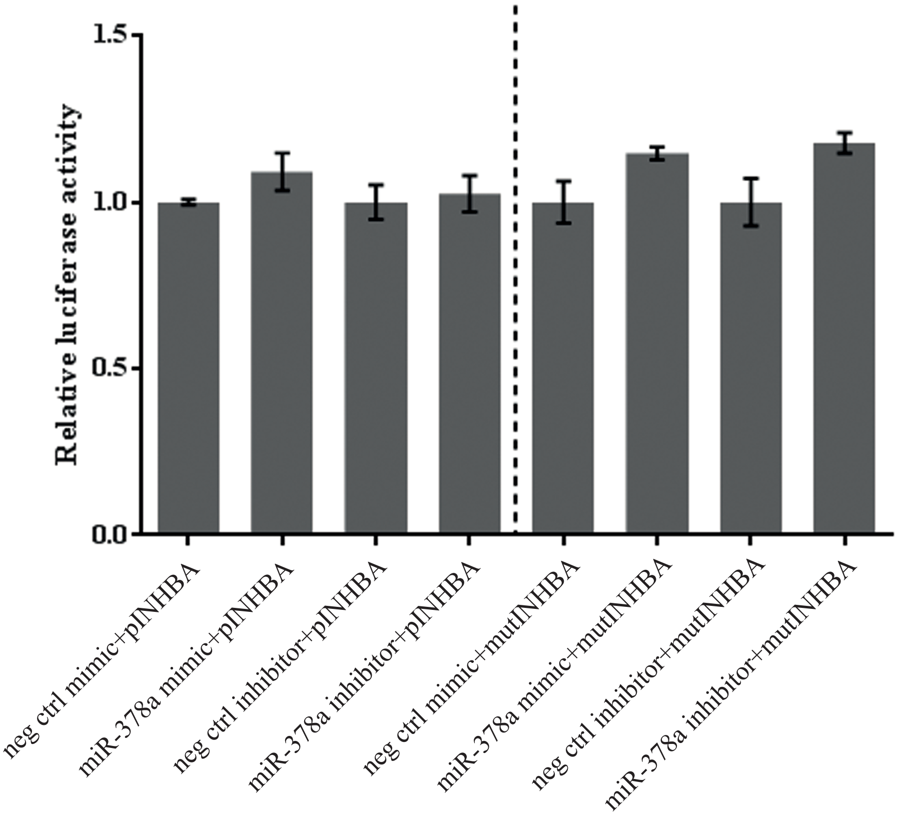

Supplementary Figure S3

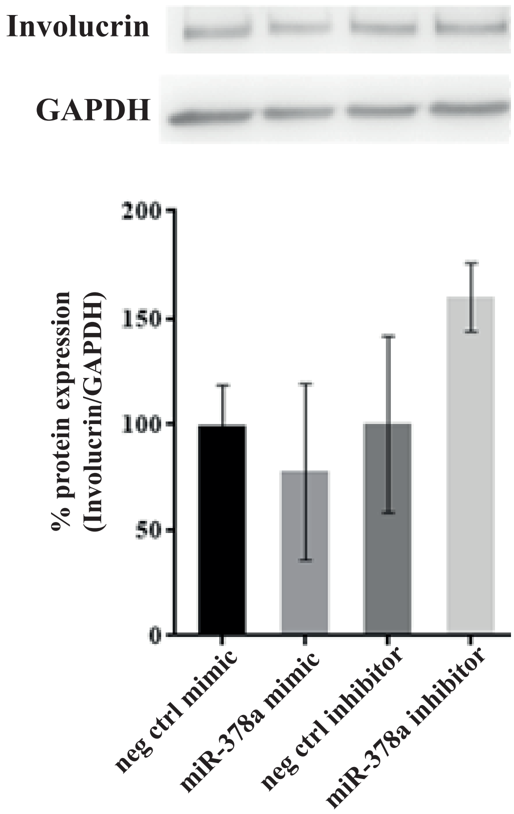

Supplementary Figure S4

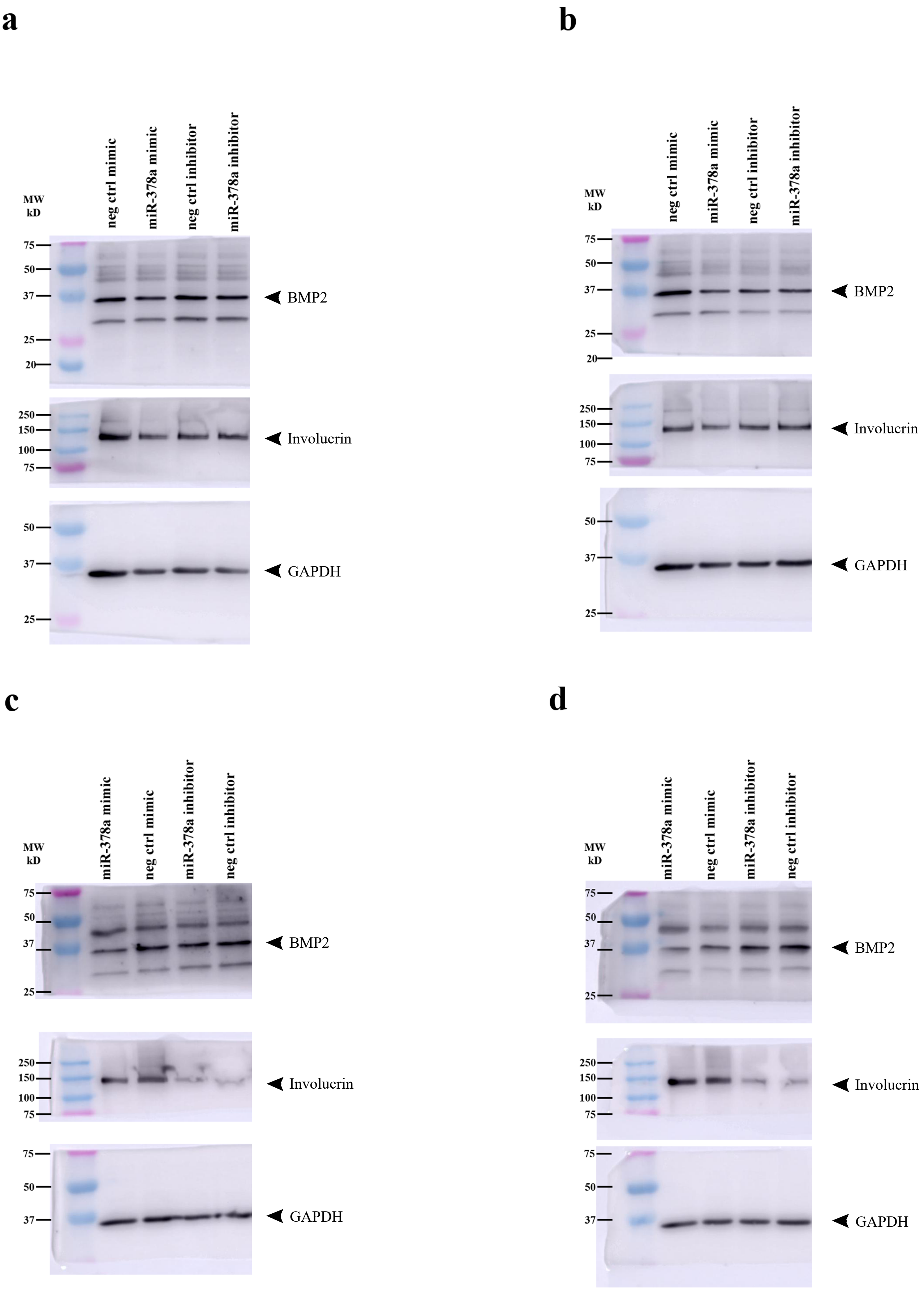

Supplementary Figure S5

Supplementary Table S1

a) Results of KEGG pathway analysis by Enrichr using DEGs from NHEK cells transfected with miR-378a mimic or its control for 24 and 48 hours.

**ID**	**Description**	**P-value**	**Gene**	**Expression**	**Time**
hsa05166	Human T-cell leukemia virus 1 infection	2.63E-02	TNF/CDC23/ETS1/SLC25A6	down	24h
hsa05217	Basal cell carcinoma	4.18E-03	LEF1/PTCH1/BMP2	down	24h
hsa04350	TGF-beta signaling pathway	1.26E-02	TNF/BMP2/INHBA	down	24h
hsa04371	Apelin signaling pathway	3.39E-02	BORCS8-MEF2B/GNA13/APLN	down	24h
hsa01523	Antifolate resistance	1.12E-02	TNF/SLC46A1	down	24h
hsa05134	Legionellosis	3.52E-02	TNF/C3	down	24h
hsa05221	Acute myeloid leukemia	4.72E-02	LEF1/PIM2	down	24h
hsa05211	Renal cell carcinoma	4.98E-02	RAP1B/ETS1	down	24h
hsa05165	Human papillomavirus infection	1.45E-02	JAG1/PPP2R1B/NRAS/ITGA11/RBPJ/THBS2/VEGFA/COL6A3/TADA3/WNT3	up	24h
hsa04014	Ras signaling pathway	3.87E-02	MET/NRAS/VEGFA/PLA2G4F/PLA2G4B/RASA3/KSR1	up	24h
hsa00564	Glycerophospholipid metabolism	2.12E-03	GPAT3/PLPP3/PLA2G4F/GPCPD1/PLA2G4B/CDS1	up	24h
hsa04370	VEGF signaling pathway	1.19E-03	NRAS/VEGFA/PLA2G4F/SH2D2A/PLA2G4B	up	24h
hsa04912	GnRH signaling pathway	8.61E-03	NRAS/MAP2K4/PLA2G4F/HBEGF/PLA2G4B	up	24h
hsa05231	Choline metabolism in cancer	1.07E-02	NRAS/PLPP3/PLA2G4F/GPCPD1/PLA2G4B	up	24h
hsa05418	Fluid shear stress and atherosclerosis	4.09E-02	MAP2K4/PLAT/VEGFA/THBD/ACVR2B	up	24h
hsa05219	Bladder cancer	2.24E-03	NRAS/TYMP/VEGFA/HBEGF	up	24h
hsa04913	Ovarian steroidogenesis	4.98E-03	CYP1B1/PLA2G4F/PLA2G4B/BMP6	up	24h
hsa04730	Long-term depression	8.85E-03	PPP2R1B/NRAS/PLA2G4F/PLA2G4B	up	24h
hsa00590	Arachidonic acid metabolism	1.05E-02	ALOX15B/PLA2G4F/PLA2G4B/GPX2	up	24h

hsa04664	Fc epsilon RI signaling pathway	1.36E-02	NRAS/MAP2K4/PLA2G4F/PLA2G4B	up	24h
hsa04917	Prolactin signaling pathway	1.50E-02	NRAS/SOCS7/ESR1/SOCS5	up	24h
hsa05120	Epithelial cell signaling in Helicobacter pylori infection	1.50E-02	PTPRZ1/MET/MAP2K4/HBEGF	up	24h
hsa04012	ErbB signaling pathway	2.84E-02	EREG/NRAS/MAP2K4/HBEGF	up	24h
hsa04350	TGF-beta signaling pathway	3.90E-02	PPP2R1B/FBN1/BMP6/ACVR2B	up	24h
hsa04666	Fc gamma R-mediated phagocytosis	4.30E-02	PLPP3/ARPC5/PLA2G4F/PLA2G4B	up	24h
hsa01522	Endocrine resistance	4.44E-02	JAG1/NRAS/HBEGF/ESR1	up	24h
hsa04061	Viral protein interaction with cytokine and cytokine receptor	4.72E-02	CXCL14/ACKR3/IL24/TNFSF10	up	24h
hsa00565	Ether lipid metabolism	2.88E-02	PLPP3/PLA2G4F/PLA2G4B	up	24h
hsa05144	Malaria	3.03E-02	MET/THBS2/LRP1	up	24h
hsa00592	alpha-Linolenic acid metabolism	4.50E-02	PLA2G4F/PLA2G4B	up	24h
hsa04110	Cell cycle	2.19E-18	CDC7/TTK/ATR/CCND2/MYC/E2F3/ABL1/E2F1/CDC25A/MCM3/RBL1/CDC45/ORC6/CCNE2/CDK1/ORC3/MCM2/TP53/BUB1B/BUB1/CDC20/CCNA2/MCM7/PKMYT1/CDC6/ESPL1/DBF4/ORC1/SKP2/PCNA/CDC23	down	48h
hsa05166	Human T-cell leukemia virus 1 infection	2.94E-04	ATR/CCND2/CRTC3/MYC/E2F3/CALR/E2F1/VAC14/CCNE2/TP53/BUB1B/CDC20/CREB5/CCNA2/TNF/ESPL1/ATF6B/CDC23/SLC25A6	down	48h
hsa03030	DNA replication	2.62E-17	RNASEH2A/RFC5/LIG1/RNASEH2C/POLE/MCM3/POLD1/POLD3/POLE2/FEN1/MCM2/PRIM2/RFC3/MCM7/POLA2/PRIM1/DNA2/PCNA	down	48h
hsa03460	Fanconi anemia pathway	1.56E-13	ATR/UBE2T/ATRIP/RAD51C/BLM/RAD51/FANCA/FANCG/BRIP1/RMI2/FANCB/RMI1/FANCM/FANCI/EME1/POLH/BRCA1/BRCA2	down	48h

hsa04218	Cellular senescence	1.01E-05	ATR/CCND2/MYC/E2F3/E2F1/LIN9/CDC25A/RBL1/CCNE2/CDK1/FOXM1/TP53/RAD9A/CALML4/CCNA2/MYBL2/LIN52/SLC25A6	down	48h
hsa05169	Epstein-Barr virus infection	2.28E-03	SAP30/CCND2/MYC/E2F3/BCL2L11/CALR/E2F1/IRAK1/IRAK4/CCNE2/LYN/TP53/CCNA2/TNF/SKP2/OAS3	down	48h
hsa03440	Homologous recombination	3.68E-12	BARD1/RAD51C/POLD1/POLD3/BLM/TOPBP1/RAD51/BRIP1/RAD54L/RAD54B/EME1/XRCC2/BRCA1/BRCA2/XRCC3	down	48h
hsa05203	Viral carcinogenesis	6.18E-03	CCND2/GRB2/VAC14/RBL1/C3/CCNE2/CDK1/LYN/TP53/CDC20/CREB5/CCNA2/HDAC3/SKP2/ATF6B	down	48h
hsa05161	Hepatitis B	5.13E-03	MYC/E2F3/E2F1/GRB2/IRAK1/IRAK4/CCNE2/TP53/CREB5/CCNA2/TNF/ATF6B/PCNA	down	48h
hsa04350	TGF-beta signaling pathway	6.16E-03	MYC/RBL1/ACVR1B/FST/TGIF2/BMP2/TNF/THSD4/INHBA	down	48h
hsa04114	Oocyte meiosis	4.07E-02	SGO1/CCNE2/CDK1/BUB1/CDC20/CALML4/PKMYT1/ESPL1/CDC23	down	48h
hsa03410	Base excision repair	1.51E-05	LIG1/POLE/POLD1/POLD3/POLE2/FEN1/NEIL3/PCNA	down	48h
hsa03420	Nucleotide excision repair	2.22E-04	RFC5/LIG1/POLE/POLD1/POLD3/POLE2/RFC3/PCNA	down	48h
hsa05222	Small cell lung cancer	1.66E-02	MYC/LAMA1/E2F3/E2F1/TRAF4/CCNE2/TP53/SKP2	down	48h
hsa05215	Prostate cancer	2.21E-02	E2F3/E2F1/GRB2/FGFR1/FGFR2/CCNE2/TP53/CREB5	down	48h
hsa03008	Ribosome biogenesis in eukaryotes	4.44E-02	RIOK1/UTP14A/WDR75/NOP56/LSG1/TCOF1/REXO5/RPP25L	down	48h
hsa03430	Mismatch repair	1.02E-05	RFC5/LIG1/POLD1/POLD3/RFC3/PCNA/EXO1	down	48h
hsa04115	p53 signaling pathway	1.49E-02	ATR/CCND2/CCNE2/CDK1/TP53/GTSE1/RRM2	down	48h

hsa03018	RNA degradation	2.22E-02	BTG3/EXOSC4/EXOSC9/HSPD1/DIS3L/DCP1A/TOB2	down	48h
hsa00250	Alanine, aspartate and glutamate metabolism	9.53E-03	CAD/ASNS/ASS1/PPAT/GPT2	down	48h
hsa05219	Bladder cancer	1.46E-02	MYC/E2F3/E2F1/TP53/MMP2	down	48h
hsa01523	Antifolate resistance	2.33E-02	SLC46A1/TYMS/TNF/DHFR	down	48h
hsa03020	RNA polymerase	2.33E-02	POLR3K/POLR1E/POLR2G/POLR2C	down	48h
hsa00450	Selenocompound metabolism	2.11E-02	SCLY/TXNRD1/CTH	down	48h
hsa04010	MAPK signaling pathway	8.34E-04	PLA2G4E/EREG/PLA2G4F/PLA2G4B/FGFR4/DUSP8/TGFA/NRAS/IL1R1/MAP2K4/RAC2/MAPK11/MAPK13/MET/NLK/EFNA3/DUSP16/MAP3K5/ERBB3/TGFB1/LAMTOR3/MAPK3/CACNB3/PDGFB/NFATC1/PRKACB/RASA1/CRK/VEGFA/DUSP6/PLA2G4A	up	48h
hsa05165	Human papillomavirus infection	9.53E-03	HLA-G/WNT10A/JAG1/NOTCH3/NRAS/TADA3/HES4/LAMC2/THBS3/THBS2/FZD7/ITGB8/PPP2R1B/WNT7B/PTGS2/ITGB6/LAMB2/PPP2R5B/ATP6V0A1/COL1A1/WNT3/DLG3/COL6A1/MAPK3/FOXO1/PRKACB/ITGB4/GSK3B/VEGFA/LAMA3	up	48h
hsa04151	PI3K-Akt signaling pathway	3.64E-02	EREG/FGFR4/TGFA/GNG2/NRAS/LAMC2/THBS3/THBS2/MET/ITGB8/PPP2R1B/ITGB6/EFNA3/TLR2/LAMB2/PPP2R5B/ERBB3/COL1A1/COL6A1/MAPK3/PDGFB/MCL1/LPAR6/ITGB4/GSK3B/IL6R/VEGFA/RPTOR/LAMA3	up	48h
hsa05206	MicroRNAs in cancer	3.60E-02	MMP9/CYP1B1/HMGA2/NOTCH3/NRAS/SERPINB5/MET/PRKCE/PTGS2/DNMT3B/EFNA3/SOCS1/PLAU/FSCN1/ERBB3/WNT3/MAPK3/HDAC4/PDGFB/MCL1/DNMT3A/TIMP3/PIM1/CRK/VEGFA/RPTOR	up	48h

hsa04014	Ras signaling pathway	1.93E-03	CALML5/PLA2G4E/PLA2G4F/PLA2G4B/FGFR4/GAB2/TGFA/PLA2G3/GNG2/NRAS/RAC2/FOXO4/MET/EFNA3/RASA3/KSR1/HTR7/MAPK3/PDGFB/PRKACB/PLD1/RASA1/CALML3/VEGFA/PLA2G4A	up	48h
hsa05205	Proteoglycans in cancer	1.70E-03	MMP9/WNT10A/NRAS/MAPK11/MAPK13/MET/FZD7/WNT7B/ANK3/PLAU/TLR2/HBEGF/ERBB3/COL1A1/TGFB1/WNT3/HPSE/MAPK3/PRKACB/SDC1/TIMP3/ESR1/VEGFA	up	48h
hsa04142	Lysosome	1.32E-04	ACP5/SORT1/SMPD1/ABCA2/CTSS/SLC17A5/CTSC/CLN3/MANBA/FUCA1/ATP6V0A1/CTSD/GBA/AP1S2/NAGLU/CTSH/AP1S3/GNPTG/CD68	up	48h
hsa05418	Fluid shear stress and atherosclerosis	3.93E-04	CALML5/MMP9/NCF2/IL1R1/PLAT/MAP2K4/IL1R2/RAC2/MAPK11/MAPK13/MAP3K5/GSTM4/PDGFB/SDC1/CALML3/VEGFA/ACVR2B/THBD/GSTA4	up	48h
hsa04510	Focal adhesion	2.38E-02	BIRC3/LAMC2/RAC2/THBS3/THBS2/MET/ITGB8/ITGB6/LAMB2/COL1A1/COL6A1/MAPK3/FYN/PDGFB/ITGB4/GSK3B/CRK/VEGFA/LAMA3	up	48h
hsa04071	Sphingolipid signaling pathway	1.55E-04	GAB2/SMPD1/NRAS/SGPP2/RAC2/MAPK11/MAPK13/PRKCE/PPP2R1B/PPP2R5B/S1PR2/MAP3K5/CTSD/MAPK3/BDKRB2/FYN/PLD1/SPTLC2	up	48h
hsa04072	Phospholipase D signaling pathway	2.18E-03	PLA2G4E/PLA2G4F/PLA2G4B/GAB2/ADCY7/NRAS/DNM3/PLPP2/PLPP1/RAPGEF3/DGKQ/MAPK3/AGPAT2/FYN/PDGFB/PLD1/LPAR6/PLA2G4A	up	48h
hsa05152	Tuberculosis	1.64E-02	CALML5/NOD2/MRC2/PLA2R1/CTSS/IRAK2/MAPK11/MAPK13/TLR2/ATP6V0A1/KSR1/CTSD/RAB7A/TGFB1/IFNGR1/MAPK3/IFNGR2/CALML3	up	48h
hsa04360	Axon guidance	1.72E-02	ABLIM3/EPHA4/SEMA4B/NRAS/SEMA3C/RAC2/MET/EFNA3/SLIT3/PLXNA3/SEMA4A/MAPK3/ROBO3/FYN/PLXNB3/RASA1/GSK3B/EPHA1	up	48h

hsa05202	Transcriptional misregulation in cancer	2.93E-02	MMP9/CEBPA/MAF/HMGA2/MMP3/PLAT/PBX1/BIRC3/IL1R2/MET/TRAF1/FUT8/PLAU/FOXO1/CDK14/MLLT3/PAX8/DUSP6	up	48h
hsa00564	Glycerophospholipid metabolism	4.06E-05	GPAT3/PLA2G4E/PLA2G4F/PLA2G4B/PLA2G3/MBOAT2/PLPP2/PLPP1/ETNK2/DGKQ/CDS1/AGPAT2/PLD1/GPCPD1/CHKB/LPGAT1/PLA2G4A	up	48h
hsa04750	Inflammatory mediator regulation of TRP channels	5.30E-05	CALML5/PLA2G4E/PLA2G4F/PLA2G4B/TRPV3/ADCY7/IL1R1/MAPK11/MAPK13/PRKCE/PRKCQ/P2RY2/BDKRB2/PRKACB/CALML3/CYP2J2/PLA2G4A	up	48h
hsa04668	TNF signaling pathway	2.25E-04	MMP9/CCL20/MMP3/JAG1/NOD2/MAP2K4/BIRC3/MAPK11/MAPK13/TRAF1/CXCL1/PTGS2/RIPK3/MAP3K5/MAPK3/CFLAR/MLKL	up	48h
hsa04915	Estrogen signaling pathway	2.50E-03	CALML5/MMP9/KRT16/TGFA/ADCY7/KRT10/NRAS/KRT19/KRT15/HBEGF/CTSD/MAPK3/PRKACB/KRT34/KRT23/CALML3/ESR1	up	48h
hsa04912	GnRH signaling pathway	7.55E-05	CALML5/PLA2G4E/PLA2G4F/PLA2G4B/ADCY7/NRAS/MAP2K4/MAPK11/MAPK13/HBEGF/MAPK3/PRKACB/PLD1/EGR1/CALML3/PLA2G4A	up	48h
hsa04933	AGE-RAGE signaling pathway in diabetic complications	1.83E-04	SERPINE1/NRAS/MAPK11/MAPK13/PRKCE/COL1A1/TGFB1/MAPK3/FOXO1/PLCD1/NFATC1/PLCD4/PIM1/EGR1/VEGFA/THBD	up	48h
hsa04217	Necroptosis	4.21E-02	PLA2G4E/PLA2G4F/PLA2G4B/TNFSF10/SMPD1/BIRC3/RIPK3/PYGB/IFNGR1/CHMP4C/CFLAR/MLKL/SPATA2L/IFNGR2/PLA2G4A	up	48h
hsa00590	Arachidonic acid metabolism	1.07E-05	ALOX15B/AKR1C3/GPX2/PLA2G4E/PLA2G4F/PLA2G4B/GGT1/GPX7/PLA2G3/PTGS2/EPHX2/GPX3/CYP2J2/PLA2G4A	up	48h
hsa05231	Choline metabolism in cancer	1.45E-03	PLA2G4E/PLA2G4F/PLA2G4B/NRAS/RAC2/PLPP2/PLPP1/DGKQ/MAPK3/PDGFB/PLD1/GPCPD1/CHKB/PLA2G4A	up	48h

hsa04726	Serotonergic synapse	6.45E-03	ALOX15B/PLA2G4E/PLA2G4F/PLA2G4B/GNG2/NRAS/PTGS2/RAPGEF3/HTR7/MAPK3/PRKACB/MAOA/CYP2J2/PLA2G4A	up	48h
hsa04068	FoxO signaling pathway	1.92E-02	TNFSF10/NRAS/MAPK11/CDKN2B/MAPK13/FOXO4/NLK/FBXO25/TGFB1/MAPK3/FOXO1/HOMER2/CCNG2/CDKN2D	up	48h
hsa04140	Autophagy - animal	2.71E-02	NRBF2/NRAS/ATG4A/ULK1/RRAGC/PRKCQ/CTSD/RAB7A/MAPK3/CFLAR/PRKACB/TP53INP2/RPTOR/WIPI1	up	48h
hsa04371	Apelin signaling pathway	2.71E-02	CALML5/JAG1/NOTCH3/SERPINE1/ADCY7/GNG2/NRAS/PLAT/PRKCE/MAPK3/HDAC4/PRKACB/EGR1/CALML3	up	48h
hsa04910	Insulin signaling pathway	2.71E-02	CALML5/NRAS/SOCS1/PTPN1/PYGB/MAPK3/FOXO1/PPP1R3B/PRKACB/CBLB/GSK3B/CRK/CALML3/RPTOR	up	48h
hsa04657	IL-17 signaling pathway	2.85E-03	MMP1/S100A8/MMP9/CCL20/S100A9/MMP3/MAPK11/MAPK13/CXCL1/PTGS2/MAPK3/GSK3B/LCN2	up	48h
hsa04666	Fc gamma R-mediated phagocytosis	3.76E-03	PLA2G4E/PLA2G4F/PLA2G4B/GAB2/GSN/RAC2/PRKCE/PLPP2/PLPP1/MAPK3/PLD1/CRK/PLA2G4A	up	48h
hsa05146	Amoebiasis	5.80E-03	SERPINB3/IL1R1/IL1R2/LAMC2/SERPINB13/CXCL1/TLR2/LAMB2/COL1A1/RAB7A/TGFB1/PRKACB/LAMA3	up	48h
hsa04722	Neurotrophin signaling pathway	1.99E-02	CALML5/SORT1/NRAS/IRAK2/MAPK11/MAPK13/MAP3K5/MAPK3/MAGED1/GSK3B/ZNF274/CRK/CALML3	up	48h
hsa04380	Osteoclast differentiation	3.39E-02	ACP5/NCF2/GAB2/IL1R1/MAPK11/MAPK13/SOCS1/TGFB1/IFNGR1/MAPK3/FYN/NFATC1/IFNGR2	up	48h

hsa04926	Relaxin signaling pathway	3.58E-02	MMP1/MMP9/ADCY7/GNG2/NRAS/MAP2K4/MAPK11/MAPK13/COL1A1/TGFB1/MAPK3/PRKACB/VEGFA	up	48h
hsa04913	Ovarian steroidogenesis	2.49E-05	AKR1C3/CYP1B1/PLA2G4E/PLA2G4F/PLA2G4B/ADCY7/PTGS2/CYP1A1/HSD17B1/PRKACB/CYP2J2/PLA2G4A	up	48h
hsa04370	VEGF signaling pathway	1.15E-04	PLA2G4E/PLA2G4F/PLA2G4B/SH2D2A/NRAS/RAC2/MAPK11/MAPK13/PTGS2/MAPK3/VEGFA/PLA2G4A	up	48h
hsa04664	Fc epsilon RI signaling pathway	4.64E-04	PLA2G4E/PLA2G4F/PLA2G4B/GAB2/NRAS/MAP2K4/RAC2/MAPK11/MAPK13/MAPK3/FYN/PLA2G4A	up	48h
hsa04512	ECM-receptor interaction	4.58E-03	LAMC2/THBS3/THBS2/ITGB8/ITGB6/NPNT/LAMB2/COL1A1/COL6A1/SDC1/ITGB4/LAMA3	up	48h
hsa01522	Endocrine resistance	1.08E-02	MMP9/JAG1/NOTCH3/ADCY7/NRAS/MAPK11/MAPK13/HBEGF/MAPK3/BIK/PRKACB/ESR1	up	48h
hsa05142	Chagas disease	1.45E-02	SERPINE1/MAP2K4/MAPK11/MAPK13/PPP2R1B/TLR2/TGFB1/IFNGR1/MAPK3/BDKRB2/CFLAR/IFNGR2	up	48h
hsa05145	Toxoplasmosis	2.83E-02	BIRC3/LAMC2/MAPK11/MAPK13/SOCS1/TLR2/LAMB2/TGFB1/IFNGR1/MAPK3/IFNGR2/LAMA3	up	48h
hsa05215	Prostate cancer	2.41E-02	MMP9/MMP3/TGFA/NRAS/PLAT/IL1R2/PLAU/MAPK3/FOXO1/PDGFB/GSK3B	up	48h
hsa04916	Melanogenesis	3.14E-02	CALML5/WNT10A/ADCY7/NRAS/FZD7/WNT7B/WNT3/MAPK3/PRKACB/GSK3B/CALML3	up	48h
hsa04974	Protein digestion and absorption	3.56E-02	COL5A3/PRSS3/SLC36A1/COL5A2/COL7A1/COL5A1/SLC7A8/COL1A1/COL6A1/COL8A2/COL22A1	up	48h
hsa04659	Th17 cell differentiation	4.51E-02	IL1R1/MAPK11/MAPK13/PRKCQ/RORA/TGFB1/IFNGR1/MAPK3/NFATC1/IFNGR2/IL6R	up	48h

hsa00480	Glutathione metabolism	1.43E-03	GPX2/GGT6/GGT1/GPX7/ODC1/GSTM4/GGT7/GPX3/OPLAH/GSTA4	up	48h
hsa04012	ErbB signaling pathway	2.45E-02	EREG/TGFA/NRAS/MAP2K4/HBEGF/ERBB3/MAPK3/CBLB/GSK3B/CRK	up	48h
hsa04658	Th1 and Th2 cell differentiation	3.96E-02	MAF/JAG1/NOTCH3/MAPK11/MAPK13/PRKCQ/IFNGR1/MAPK3/NFATC1/IFNGR2	up	48h
hsa05323	Rheumatoid arthritis	4.22E-02	MMP1/ACP5/CCL20/MMP3/CXCL1/TLR2/ATP6V0A1/TGFB1/TNFSF13/VEGFA	up	48h
hsa05211	Renal cell carcinoma	1.74E-02	TGFA/NRAS/MET/EGLN3/TGFB1/MAPK3/PDGFB/CRK/VEGFA	up	48h
hsa01524	Platinum drug resistance	2.44E-02	ATP7B/BIRC3/MAP3K5/GSTM4/ERCC1/MAPK3/SLC31A1/ATP7A/GSTA4	up	48h
hsa05140	Leishmaniasis	3.32E-02	NCF2/MAPK11/MAPK13/PTGS2/TLR2/TGFB1/IFNGR1/MAPK3/IFNGR2	up	48h
hsa01521	EGFR tyrosine kinase inhibitor resistance	3.83E-02	TGFA/NRAS/MET/ERBB3/MAPK3/PDGFB/GSK3B/IL6R/VEGFA	up	48h
hsa00565	Ether lipid metabolism	6.61E-03	PLA2G4E/PLA2G4F/PLA2G4B/PLA2G3/PLPP2/PLPP1/PLD1/PLA2G4A	up	48h
hsa00140	Steroid hormone biosynthesis	2.36E-02	AKR1C3/CYP1B1/CYP1A1/STS/SULT2B1/AKR1C2/HSD17B1/AKR1C1	up	48h
hsa00561	Glycerolipid metabolism	2.36E-02	PNLIPRP3/GPAT3/DGAT2/MBOAT2/PLPP2/PLPP1/DGKQ/AGPAT2	up	48h
hsa05321	Inflammatory bowel disease	3.32E-02	MAF/NOD2/TLR2/RORA/TGFB1/IFNGR1/NFATC1/IFNGR2	up	48h
hsa04137	Mitophagy - animal	4.18E-02	NRAS/BNIP3L/PINK1/ULK1/TBC1D17/TFEB/RAB7A/MFN1	up	48h
hsa04917	Prolactin signaling pathway	4.84E-02	NRAS/MAPK11/MAPK13/SOCS1/SOCS5/MAPK3/GSK3B/ESR1	up	48h
hsa05120	Epithelial cell signaling in Helicobacter pylori infection	4.84E-02	PTPRZ1/MAP2K4/MAPK11/MAPK13/MET/CXCL1/HBEGF/ATP6V0A1	up	48h

hsa05219	Bladder cancer	8.47E-03	MMP1/TYMP/MMP9/NRAS/HBEGF/MAPK3/VEGFA	up	48h
hsa04975	Fat digestion and absorption	1.10E-02	DGAT2/PLA2G3/PLPP2/SLC27A1/PLPP1/SLC27A4/AGPAT2	up	48h
hsa02010	ABC transporters	1.40E-02	ABCA12/ABCA2/DEFB1/ABCG1/ABCD1/ABCG2/ABCC5	up	48h
hsa00600	Sphingolipid metabolism	2.18E-02	SMPD1/SGPP2/PLPP2/PLPP1/GBA/UGCG/SPTLC2	up	48h
hsa05144	Malaria	2.41E-02	LRP1/THBS3/THBS2/MET/TLR2/TGFB1/SDC1	up	48h
hsa00592	alpha-Linolenic acid metabolism	2.52E-03	PLA2G4E/PLA2G4F/PLA2G4B/PLA2G3/ACAA1/PLA2G4A	up	48h
hsa00591	Linoleic acid metabolism	5.56E-03	PLA2G4E/PLA2G4F/PLA2G4B/PLA2G3/CYP2J2/PLA2G4A	up	48h
hsa00430	Taurine and hypotaurine metabolism	2.25E-02	GGT6/GGT1/GGT7	up	48h

b) Results of KEGG pathway analysis by Enrichr using DEGs from NHEK cells transfected with miR-378a inhibitor or its control for 24 and 48 hours.

**ID**	**Term**	**P-value**	**Gene**	**Expression**	**Time**
hsa00830	Retinol metabolism	2.51E-02	CYP26B1	down	24h
hsa04061	Viral protein interaction with cytokine and cytokine receptor	3.67E-02	ACKR3	down	24h
hsa04668	TNF signaling pathway	4.99E-04	BIRC3/CXCL5/NOD2/CSF2/PTGS2	up	24h
hsa05167	Kaposi sarcoma-associated herpesvirus infection	5.51E-03	HLA-G/C3/CSF2/PTGS2/PREX1	up	24h
hsa04060	Cytokine-cytokine receptor interaction	2.99E-02	IL1R2/INHBA/CXCL5/CSF2/IL32	up	24h
hsa04657	IL-17 signaling pathway	2.26E-03	CXCL5/CSF2/MMP1/PTGS2	up	24h
hsa04530	Tight junction	1.76E-02	CGN/PARD6B/CLDN7/DLG1	up	24h

hsa05202	Transcriptional misregulation in cancer	2.67E-02	BIRC3/IL1R2/CSF2/PLAT	up	24h
hsa05166	Human T-cell leukemia virus 1 infection	4.04E-02	IL1R2/HLA-G/CSF2/DLG1	up	24h
hsa04612	Antigen processing and presentation	1.10E-02	HSPA6/CTSS/HLA-G	up	24h
hsa04610	Complement and coagulation cascades	1.38E-02	C3/SERPINB2/PLAT	up	24h
hsa05323	Rheumatoid arthritis	1.76E-02	CXCL5/CSF2/MMP1	up	24h
hsa05150	Staphylococcus aureus infection	1.91E-02	KRT19/KRT13/C3	up	24h
hsa05146	Amoebiasis	2.24E-02	IL1R2/CSF2/SERPINB9	up	24h
hsa04915	Estrogen signaling pathway	4.83E-02	HSPA6/KRT19/KRT13	up	24h
hsa05418	Fluid shear stress and atherosclerosis	4.92E-02	IL1R2/HMOX1/PLAT	up	24h
hsa00480	Glutathione metabolism	4.49E-02	CHAC1/GGT6	up	24h
hsa05134	Legionellosis	4.49E-02	HSPA6/C3	up	24h
hsa04978	Mineral absorption	4.78E-02	SLC5A1/HMOX1	up	24h
hsa04060	Cytokine-cytokine receptor interaction	3.08E-04	IL33/TNFSF10/BMP4/CXCL14	down	48h
hsa05130	Pathogenic Escherichia coli infection	1.51E-03	MAPK11/TNFSF10/TUBA4A	down	48h
hsa05132	Salmonella infection	2.96E-03	MAPK11/TNFSF10/TUBA4A	down	48h
hsa04061	Viral protein interaction with cytokine and cytokine receptor	6.40E-03	TNFSF10/CXCL14	down	48h
hsa04722	Neurotrophin signaling pathway	8.97E-03	MAPK11/TP73	down	48h
hsa04611	Platelet activation	9.71E-03	PTGS1/MAPK11	down	48h
hsa04068	FoxO signaling pathway	1.08E-02	MAPK11/TNFSF10	down	48h
hsa04210	Apoptosis	1.16E-02	TNFSF10/TUBA4A	down	48h

hsa05418	Fluid shear stress and atherosc lerosis	1.21E-02	MAPK11/BMP4	down	48h
hsa04550	Signaling pathways regulating pluripotency of stem cells	1.28E-02	MAPK11/BMP4	down	48h
hsa04261	Adrenergic signaling in cardio myocytes	1.40E-02	MAPK11/SCN4B	down	48h
hsa04390	Hippo signaling pathway	1.53E-02	BMP4/TP73	down	48h
hsa04217	Necroptosis	1.56E-02	IL33/TNFSF10	down	48h
hsa05164	Influenza A	1.79E-02	IL33/TNFSF10	down	48h
hsa05020	Prion disease	4.28E-02	MAPK11/TUBA4A	down	48h
hsa04530	Tight junction	7.05E-04	PARD6B/CGN/CLDN7/OCLN/NEDD4L/CLDN4	up	48h
hsa05418	Fluid shear stress and atherosc lerosis	1.91E-03	IL1R2/HMOX1/ASS1/SQSTM1/EDN1	up	48h
hsa04060	Cytokine-cytokine receptor interaction	4.03E-02	IL1RL1/CSF2/INHBA/IL1R2/TNFSF15	up	48h
hsa04657	IL-17 signaling pathway	3.04E-03	CSF2/MMP1/PTGS2/MMP3	up	48h
hsa04668	TNF signaling pathway	5.69E-03	CSF2/PTGS2/MMP3/EDN1	up	48h
hsa04514	Cell adhesion molecules	1.52E-02	CLDN7/OCLN/HLA-G/CLDN4	up	48h
hsa05202	Transcriptional misregulation in cancer	3.46E-02	CSF2/MMP3/IL1R2/GADD45B	up	48h
hsa05167	Kaposi sarcoma-associated herpesvirus infecti on	3.51E-02	CSF2/PTGS2/FGF2/HLA-G	up	48h
hsa05416	Viral myocarditis	6.65E-03	CD55/HLA-G/ABL2	up	48h
hsa01230	Biosynthesis of amino acids	1.23E-02	ASNS/CBS/ASS1	up	48h
hsa05323	Rheumatoid arthritis	2.18E-02	CSF2/MMP1/MMP3	up	48h
hsa04640	Hematopoietic cell lineage	2.56E-02	CSF2/IL1R2/CD55	up	48h
hsa04670	Leukocyte transendothelial mi gration	3.67E-02	CLDN7/OCLN/CLDN4	up	48h

hsa04977	Vitamin digestion and absorpt ion	1.03E-02	LRAT/SLC19A2	up	48h
hsa00601	Glycosphingolipid biosynthes is - lacto and neolacto series	1.29E-02	FUT3/GCNT2	up	48h
hsa00250	Alanine, aspartate and glutam ate metabolism	2.35E-02	ASNS/ASS1	up	48h
hsa04216	Ferroptosis	2.84E-02	HMOX1/SLC7A11	up	48h

Supplementary Table S2

List of primers used for real-time qPCR in this study.

Gene	Forward sequence (5′ -- 3′)	Reverse sequence (5′ -- 3′)
BMP2	CCCACGGAGGAGTTTATCAC	GAGTTGGCTGTTGCAGGTTT
INHBA	GGAGAACGGGTATGTGGAGA	ACAGGTCACTGCCTTCCTTG
KIAA1522	AAACTGAACAAGGGTGGCTG	TAGGATGTGGTCTGACTGCA
KRT80	ACTCAGCCATCTTCGACCTC	ACTCCTCAACCTTCTCCAGC
PEF1	AGCCACCTCCAAGTTCCTAC	AGCCACTGTGATCTGAGTCC
TMEM245	TGGAAGGAGCAATCATCGGT	GTTTGGCGTGGGAACTGAAT
TOB2	GAAGGGAGCTGTGAAAGTGC	GAATGAAGGTAGGGCTGGGT
GUSB	TGCAGGTGATGGAAGAAGTG	TTGCTCACAAAGGTCACAGG

Supplementary Table S3

List of miR-378a, *BMP2* 3’ UTR, *BMP2* 3’ UTR mutant, *INHBA* 3’ UTR and *INHBA* 3’ UTR mutant used in the luciferase study.

Name	Sequence
has-miR-378a	3’ CGGAAGACTGAGGTTCAGGTCA 5’
BMP2 3’UTR	5’ AGTTCACAAGTTCAAGTCCAGA 3’
BMP2 3’UTR mutant	5’ AGTTCACAAGTTCAAGTCTCA 3’
INHBA 3’UTR	5’ AGCTTGAGAGTGGATTCTAATCCAGG 3’
INHBA 3’UTR mutant	5’ AGCTTGAGAGTGGATTCTAATGTCG 3’
